# Exploring propolis-derived compounds as quorum sensing inhibitors for *Candida albicans*: a molecular docking and dynamics simulations study

**DOI:** 10.1038/s41598-025-18001-1

**Published:** 2025-09-25

**Authors:** Fettouma Chraa, Doha EL Meskini, Ilham Kandoussi, Abdelhakim Bouyahya, Long Chiau Ming, Jactty Chew, Said Moshawih, Rachid El Jaoudi, Mouna Ouadghiri, Tarik Aanniz

**Affiliations:** 1 Medical Biotechnology Laboratory (MedBiotech), Bioinova Research Center, Medical and Pharmacy School, Mohammed V University, Rabat, Morocco; 2 Laboratory of Human Pathologies Biology, Faculty of Sciences, Mohammed Vth University in Rabat, Rabat, Morocco; 3 Datta Meghe College of Pharmacy, Datta Meghe Institute of Higher Education and Research (deemed to be a University), Sawangi, Wardha, Maharashtra India; 4 School of Pharmacy, Faculty of Medical and Life Sciences, Sunway University, Sunway City, Malaysia; 5 Sir Jeffrey Cheah Sunway Medical School, Faculty of Medical and Life Sciences, Sunway University, Sunway City, 47500 Malaysia; 6 Faculty of Pharmacy, Al-Ahliyya Amman University, Amman, Jordan

**Keywords:** Resistance, Quorum quenching, Computer-aided drug design, Structure-based virtual screening, Biofilm, Developmental biology, Drug discovery

## Abstract

The opportunistic fungal pathogen *Candida albicans* (*C. albicans*) employs quorum sensing (QS) to regulate virulence factors such as biofilm formation and morphological switching. Targeting QS offers a promising approach to attenuate pathogenicity without promoting resistance. The objective of the present study was to computationally screen a library of 106 propolis-derived compounds to identify natural quorum-sensing inhibitors targeting the CYC and RAS1 receptors. Advanced techniques, including molecular docking, molecular dynamics (MD) simulations, and ADMET predictions, were employed to establish a workflow for structure-based virtual screening. Farnesol, used as a reference compound, showed a good binding affinity (-7.0 kcal/mol) toward CYC and RAS1 receptors. Two propolis flavonoids, kaempferol-3-O-4-O-p-coumaryl-glucoside (KCG) and isorhamnetin-3-glucoside-7-rhamnoside (IGR), exhibited stronger binding affinities with CYC and RAS1 receptors (between − 8.4 and − 10.3 kcal/mol). Based on Prime MMGBSA binding free energy calculations, the RAS1-KCG complex demonstrated the most favorable interaction, driven by significant coulombic and Van der Waals contributions. The CYC-KCG complex also showed relatively strong binding. These results highlight the potential of KCG as a versatile inhibitor capable of interacting with both targets. MD simulations further confirmed the excellent conformational stability of both KCG-receptor complexes, as indicated by low RMSD values, suggesting favorable dynamic behavior. These findings provide a strong foundation for subsequent validation.

**Supplementary Information:**

The online version contains supplementary material available at 10.1038/s41598-025-18001-1.

## Introduction

The discovery and naming of *C. albicans* span from ancient Greek scholars to modern research, reflecting the evolution of our understanding of *Candida* species. Although originally thought to be an exudate found in human hosts, it was later identified as a pathogenic microorganism^1^. The earliest known manifestation of candidiasis, oral thrush, has been documented in medical literature for nearly 200 years^2^. Clinically, thrush appears as whitish plaques on the tongue, buccal mucosa, or oropharynx. Whether thrush originated internally from the host, externally as an infectious agent, or as a combination of both was a hotly debated topic in the scientific community^3^.

Over the past 20 years, *C. albicans* has emerged as a prevalent nosocomial pathogen and a major public health concern^4^. As a commensal yeast, it normally inhabits the female reproductive tract, oral cavity, skin, and gastrointestinal tract without causing harm^5^. However, in immunocompromised individuals, it can translocate from mucosal sites, such as the gut, into the bloodstream, leading to systemic infection^6^. Moreover, *C. albicans* forms tough biofilms on both abiotic surfaces and within host tissues. These biofilms comprise multiple cellular morphologies, including oval pseudohyphal cells, elongated hyphal cells, and round budding yeast cells embedded in a protective extracellular matrix^7^.

In the quest to discover or develop effective potent anticandidal agents, scientific focus has increasingly shifted toward both synthetic compounds and/or naturally occurring plant-derived molecules. Notably, several substances have shown potent inhibitory effects on hyphal formation, adhesion, and biofilm development, including purpurin, nepodin, and coumarin. These compounds disrupt key signaling pathways, including the cAMP-PKA and MAPK pathways, which regulate the yeast-to-hyphae transition^8^. QS is increasingly recognized in the scientific literature as a mechanism of microbial signaling and intercellular communication. Mukherjee and his team demonstrated that QS enables bacteria to coordinate complex group behaviors, such as biofilm formation, production of virulence factors, and antibiotic resistance, particularly in extremely heterogeneous environments like multi-species communities and host-associated biofilms. These findings underscore the pivotal role of QS in bacterial adaptability and pathogenicity^9^.

QS, also referred to as auto-induction, is a phenomenon in which individual cells produce and release small amounts of diffusible signaling molecules called autoinducers (AIs) into the environment, which are subsequently detected by all cells within the population. QS also plays a pivotal role in governing morphogenetic changes in *C. albicans*, particularly the transition from yeast to filamentous forms, both of which are associated with virulence. According to Kruppa, QS in *C. albicans* is essential for population-level coordination and environmental adaptation, enhancing stress responses and promoting biofilm formation^10,11^. The primary QS molecules (QSMs) found in *C. albicans* include tryptophol and phenylethyl alcohol. Tyrosol, farnesoic acid, and farnesol are the three other QSMs that have been discovered in recent years. Notably, farnesoic acid has been reported in only one strain of *C. albicans* and exhibits lower activity than farnesol^12^. Farnesol remains the most extensively studied QSM and has been shown to inhibit the yeast-to-hyphae transition at high cell densities and under conditions that typically promote filamentation^10^. Han et al. reported that the accumulation of farnesol and tyrosol is directly influenced by environmental conditions and metabolic pathways, providing feedback control for cellular differentiation through gene regulation. Under nutrient-limited conditions, tyrosol promotes germ tube formation, whereas farnesol acts as a negative regulator of filamentation by interfering with the Ras1-cAMP-PKA signaling pathway. Farnesol was also found to suppress the expression of HST7 and CPH1, thereby inhibiting MAP kinase signaling cascades. Furthermore, farnesol downregulates the MAP kinase and cAMP-PKA pathways while upregulating hyphal suppressor genes such as TUP1 and HOG1, ultimately preventing morphogenesis in *C. albicans*^13^. In contrast, tyrosol has been shown to induce hyphal development during the early and intermediate stages of biofilm formation. Therefore, QSMs modulate *C. albicans* morphogenesis both positively and negatively in a cell density-dependent manner^14^. Given the increasing challenge of eliminating *C. albicans* biofilms and the growing concern over antifungal resistance, finding novel therapeutic strategies is imperative. Consequently, targeting the QS system represents a valuable option^15^.

Additionally, researchers have rapidly and cost-effectively adopted the strategy of repurposing FDA-approved drugs for anti-QS and antimicrobial discovery based on the Computer Aided Drug Design (CADD) approach by targeting the QS system. Hence, fexofenadine, ivermectin, nitrofurantoin, levocetirizine, atorvastatin, and aceclofenac have demonstrated significant in vitro anti-QS and antivirulence activity through strong docking-based interactions with QS receptors^16^. Additional repurposed drugs such as metformin, propranolol, and amitriptyline have shown antibacterial activity against *Bacillus pumilus*, *Pseudomonas aeruginosa*, and *Staphylococcus aureus*, displaying attractive binding affinities for multiple bacterial targets^17^. Ketoprofen has also been shown to reduce virulence and biofilm formation by mimicking natural ligands of the QS regulator PqsR^18^. Other drugs, like hydralazine, ambroxol, albendazole, dimetridazole, and ribavirin, have demonstrated interference with biofilm formation and the QS signaling system. Recently, Spaggiari et al.^19^ conducted a comprehensive analysis of patents and patent applications from 2019 to 2023 focused on the use of QS as a valuable target for antimicrobial design and discovery. Collectively, these findings support the therapeutic potential of repurposed drugs as antivirulence agents capable of attenuating pathogenicity without promoting resistance^20–28^. However, despite significant advances in bacterial systems, QS-targeted drug repurposing remains an underexplored area in antifungal research, particularly against pathogens like *C. albicans*. This emphasizes the need to screen and investigate natural product-based alternatives.

Propolis is a naturally occurring resinous substance known for its wide range of pharmacological properties. It typically consists of 50% resin, 30% wax, 10% essential oils, 5% pollen, and other components (5%), such as minerals, debris, and various bioactive molecules^29^. Its chemical composition is highly complex and influenced by several factors, including honeybee species, surrounding plant sources, collection methods, geographical and climatic variations, seasonal timing, and exposure to light^30^. Propolis has demonstrated a broad spectrum of biological activities, including anticancer, spasmolytic, antioxidant, antidiabetic, hepatoprotective, cardioprotective, and immunomodulatory effects^31^. Additionally, researchers have evaluated propolis extract for its ability to reduce the pathogenicity of infectious agents ^32,33^. The identified compounds in propolis are categorized into many chemical classes, such as volatile oils, aromatic acids, amino acids, terpenoids, fatty acids, flavonoids, phenols, and others^34^. These groups contain a variety of active molecules, including kaempferol, pinocembrin, luteolin, chrysin, coumaric acid, and ferulic acid, among others^35^.

RAS1 and CYC are central regulators of QS and morphogenesis in *C. albicans*. Targeting these proteins may disrupt the pathogen’s communication and coordination mechanisms. This approach introduces a novel concept in antifungal therapy, disarming the fungus rather than destroying it. This strategy offers the potential to reduce resistance development and enhance clinical outcomes in the management of fungal infections. Despite its potential, Moroccan propolis remains underexplored. Furthermore, pure compounds from Moroccan propolis that specifically target the key QS regulators RAS1 and CYC in *C. albicans* have not been thoroughly evaluated before. Our study addresses this gap by identifying promising propolis-derived QS inhibitors through the CADD approach, which combines molecular docking and dynamics simulations (MD) as well as the ADMET predictions. This integrated approach represents an innovative strategy to attenuate fungal virulence while minimizing the risk of resistance emergence.

## Materials and methods

**Fig. 1:**
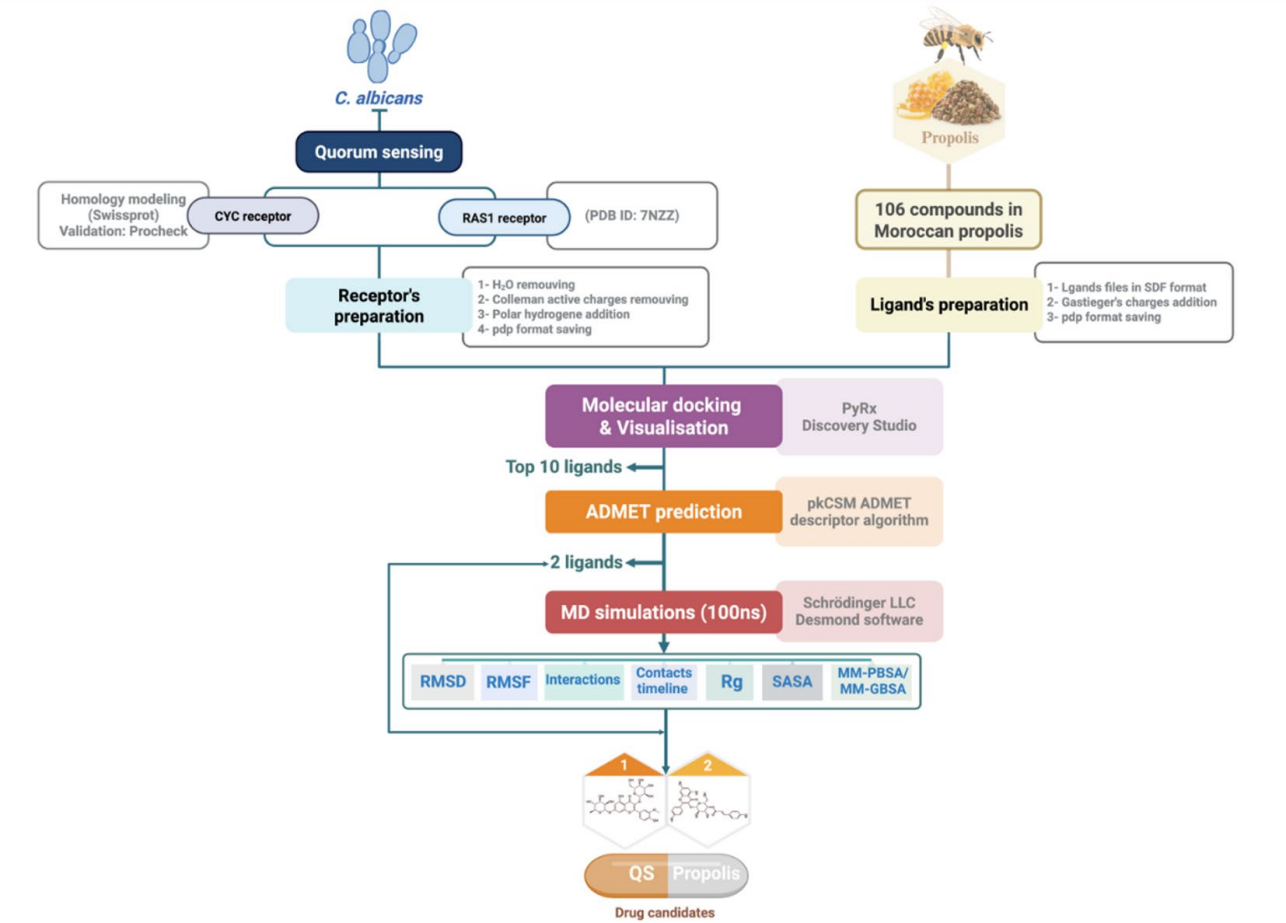
Computational workflow for screening Moroccan propolis compounds as potential inhibitors of *C. albicans* QS.

The workflow depicted in the diagram outlines the process used to identify potential QS inhibitors from Moroccan propolis against *C. albicans* (Fig. 1). The research began with the selection of two key QS receptors, CYC and RAS1, both of which play critical roles in virulence and biofilm formation. Receptor preparation involved homology modeling and docking optimization. A library of 106 Moroccan propolis compounds was screened using molecular docking *via* PyRx software, which identified the top 10 ligands based on binding affinity. The most promising candidates were then evaluated for their drug-likeness and pharmacokinetic properties using ADMET prediction through the pKCSM algorithm. The top two ligands, along with farnesol, were subsequently subjected to a 100 ns MD simulation using Schrödinger’s Desmond software to assess their stability and interaction profiles. The MD output included critical analyses, such as root mean square deviation (RMSD), root mean square fluctuation (RMSF), radius of gyration (rGyr), solvent accessible surface area (SASA), and molecular mechanics/generalized Born surface area (MM-GBSA) calculations. These analyses facilitated the identification of the most promising QS inhibitor candidate.

### Structure generation

#### Homology modeling of CYC protein

Since the experimentally solved 3D structure of the target protein CYC is not available in public databases, homology modeling was performed using SWISS-MODEL (https://swissmodel.expasy.org/interactive) to generate a reliable structural model^36,37^. The primary sequence of CYC (Q9P977_CANAX) was retrieved from the UniProt Knowledgebase (UniProtKB) in FASTA format (https://www.uniprot.org/). This sequence was submitted to PDB-BLAST to identify suitable templates for modeling. The 3D structure of CYC was then successfully generated using SWISS-MODEL based on homology with available structural templates^38^ (Fig. 1).

#### Validation of CYC model

The stereochemical properties and accuracy of the modeled structure were evaluated using PROCHECK. This evaluation included the assessment of backbone torsion angles through Ramachandran plot analysis, analysis of main-chain geometry parameters (omega and zeta angles), and G-factor calculations for both backbone and side-chain dihedral angles. Chi1-Chi2 plots were generated to evaluate the side chain conformations of rotatable residues. Aromatic and planar residues were tested for planarity through RMS deviations from ideal geometry. Non-bonded interaction analysis was also performed to identify steric clashes or unfavorable contacts. All stereochemical parameters were evaluated according to commonly accepted threshold values^39^ (Fig. 1).

#### RAS1 protein collection

The 3D structure of the RAS1 protein (PDB ID: 7NZZ) was retrieved from the Protein Data Bank (PDB) (https://www.rcsb.org/structure/7NZZ). Prior to molecular docking studies, the protein was carefully prepared using AutoDockTools (Fig. 1).

### Data collection and ligand preparation

A local database was created containing 106 propolis molecules, which were previously identified and characterized by Belmehdi et al.^40^ for their diverse biological activities. To identify potential lead compounds capable of inhibiting the RAS1 and CYC receptors that regulate QS in *C. albicans*, this database was screened^41^. Ligand files in SDF format were retrieved from the PubChem database (https://pubchem.ncbi.nlm.nih.gov) and prepared using AutoDockTools^42^. The preparation included the addition of Gasteiger charges and non-polar hydrogens, as well as the determination of ligand flexibility to explore the different conformations the ligand can adopt during docking with the protein target, followed by saving the structures in PDB format^43^ (Fig. 1).

### Receptors preparation

Both proteins, CYC and RAS1, were prepared using AutoDockTools. Water molecules were removed, and united Kollman atomic charges and polar hydrogens were added, followed by saving the structures in PDB format for further analysis. These steps were performed according to standard computational procedures to ensure structural integrity^44–47^ (Fig. 1).

### Molecular docking protocol

Molecular docking is a computational modeling technique used to predict the preferred binding orientation of a ligand to a receptor when the two combine to form a stable complex^48^. Docking was conducted using Python Prescription (PyRx v0.8) (https://pyrx.sourceforge.io/), which incorporates AutoDock Vina. Ligand structures were imported into PyRx and converted to PDBQT format using the Open Babel tool integrated within PyRx. All PyRx parameters were kept at their default values. Virtual screening was carried out to identify potential inhibitors targeting the active sites of the RAS1 and CYC proteins. After importing the energy-minimized protein and ligand structures, blind docking was performed^49^. A grid box was generated around the entire target protein^50^. The binding affinities of the ligands to the active site were expressed in kcal/mol. The best-docked complexes were visualized using Discovery Studio^51^ (Fig. 1).

### MM-GBSA free energy calculations

As a post-docking validation procedure, Molecular Mechanics with Generalized Born and Surface Area (MM-GBSA) calculations were used. This method combines molecular mechanics (MM) energies, solvation energies from the Generalized Born (GB) model, and non-polar surface area contributions to estimate the binding affinity^52,53^. Energy calculations were performed using the Prime MM-GBSA method integrated within the Maestro module of the Schrödinger suite, utilizing OPLS_2005 (Optimized Potentials for Liquid Simulations) force field. All computations were performed using default parameters. The binding free energy is determined using the equation:

$$\Delta {{\text{G}}_{{\text{bind}}}}={\text{ }}{{\text{G}}_{{\text{complex}}}} - {\text{ }}{{\text{G}}_{{\text{protein}}}} - {\text{ }}{{\text{G}}_{{\text{ligand}}}}$$

In this equation, G_complex_ refers to the free energy of the ligand-protein complex, G_protein_ represents the energy of the receptor, and G_ligand_ represents the energy of the unbound ligand.

### Physiochemical, drug-likeness evaluation, and ADME-Tox prediction

The physiochemical properties and drug-likeness of the selected compounds were evaluated, including assessment based on Lipinski’s Rule of Five. Virtual screening results were categorized according to binding energies, with the most negative values indicating the strongest binding affinities. Then, the pharmacokinetic properties of the potential inhibitors identified through molecular docking simulations were evaluated^54^. SMILES structures of the ligands were retrieved from the PubChem database and inputted into the pkCSM ADMET descriptor algorithm, a freely accessible web service (https://biosig.lab.uq.edu.au/pkcsm/)^55^ (Fig. 1).

### MD simulations

MD simulations were performed for 100 ns using Schrödinger LLC Desmond software, version 2019-4 (https://www.schrodinger.com/platform/products/desmond/)^56^. The molecular system was solvated using the TIP3P (Transferable Intermolecular Potential 3 Points) water model within an orthorhombic box. Simulations were conducted under an NPT ensemble, maintaining a constant temperature of 300 K using a Hoover thermostat and a pressure of 1 atm using a barostat. The OPLS_2005 force field was used to model all atoms in the system^57^. Counter-ions and 0.15 M sodium chlorides were added to neutralize the system and mimic physiological conditions, respectively (Fig. 1).

### 3D superposition of protein structures and ligand-protein complexes

Three-dimensional (3D) structural superposition of protein and protein-ligand complexes was performed using UCSF Chimera software (version 1.19.0), a well-established molecular visualization and analysis tool widely used for structural alignment and comparison^58^. Representative structures at 0 ns (initial frame) and 100 ns (final frame) were extracted from MD trajectories in PDB format. The structures (both the proteins alone and protein-ligand complexes) were loaded into Chimera. The Matchmaker tool was used to align the final structure (100 ns) with the reference structure (0 ns). After the alignment, proteins were colored distinctly for CYC 3D superposition (0 ns: hot pink; 100 ns: cornflower blue) and RAS1 protein superposition (0 ns: blue; 100 ns: red). Ligands from both states were represented as molecular surfaces colored separately to highlight conformational or positional variations for the IGR ligand superposition (0 ns: yellow; 100 ns: green) and for the KCG ligand superposition (0 ns: orange; 100 ns: forest green). The resulting 3D superposition images were saved in PNG format using Chimera (Fig. 9) .

## Results

### 3D structure model of CYC

Homology modeling was performed to predict the 3D structure of the CYC receptor. This process typically involves four key steps: (i) identifying evolutionarily related proteins with experimentally determined structures to serve as templates for modeling the target protein; (ii) aligning the target sequence with template structure (s) using sequence alignment methods, with manual adjustments if necessary; (iii) constructing a three-dimensional model based on the sequence alignment; and (iv) evaluating the quality of the final model^59,60^.

The primary sequence of CYC (Q9P977_CANAX) was retrieved from UniProtKB in FASTA format. It was then submitted to PDB-BLAST to identify suitable structural templates from the PDB by aligning the target sequence with experimentally resolved protein structures showing significant sequence similarity. Once high-quality templates with appropriate sequence identity and coverage were identified, modeling was performed using the SWISS-MODEL workspace, an automated platform for comparative protein structure prediction. SWISS-MODEL generated 3D models of CYC by aligning its primary sequence with the selected template and generating models based on structural homology. The generated models were then refined and validated to ensure structural accuracy. This process yielded reliable 3D models suitable for downstream applications such as molecular docking and virtual screening.

The quality of the generated protein model was evaluated using a Ramachandran plot *via* Procheck. The predicted 3D structure of CYC showed 92.6% of residues in the most favored regions, 7.4% in additional allowed regions, and no residues in generously allowed or disallowed regions. A Ramachandran plot with 90% of residues in the most favored regions is considered as reliable as experimentally determined crystal structures^39^. The absence of residues in the disallowed regions confirmed that the models generated by SWISS-MODEL are accurate and suitable for further molecular docking studies. Stereochemical evaluation of the 3D model was performed using PROCHECK. Analysis of the main geometric parameters revealed excellent peptide bond planarity (standard deviation of the omega angle at 3.9°), absence of unfavorable non-bonded contacts, and minimal tetrahedral distortion (zeta = 0.9°). In addition, the overall G-factor was + 0.3, exceeding the expected average value and indicating overall favorable stereochemistry. RMS deviations from ring planarity reflect that aromatic residues maintained conformational planarity, while the Chi1–Chi2 scatter plots indicated that side chains adopted energetically favorable conformations. Finally, residual analysis revealed a small fraction of notable changes in internal torsional angles. The detailed PROCHECK output, including the Ramachandran plot and stereochemical evaluation, is provided in the Supplementary Data. Altogether, these findings confirm the structural integrity of the model and its compliance with established stereochemical parameters for protein structures.

### Virtual screening of propolis molecules and visualization

**Fig. 2:**
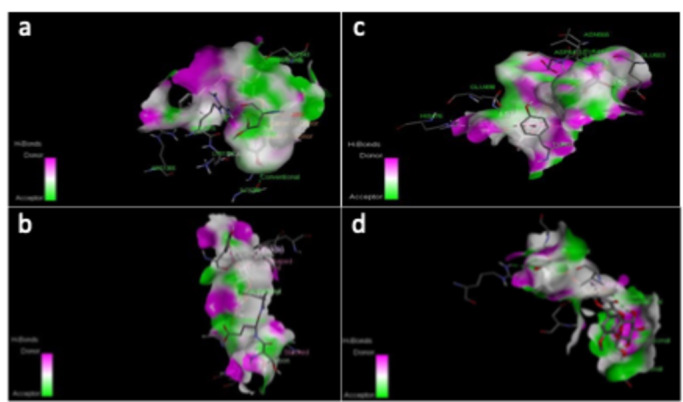
3D visualization of ligand-receptor interactions models. (a) CYC-KCG complex, (b) RAS1-KCG complex, (c) CYC-IGR complex, (d) RAS1-IGR complex. The molecular surfaces represent the hydrogen bond donor (prurple) and acceptor (green) properties of the ligand interacting with key amino acid residues within the quorum sensing receptors.

Farnesol, the natural ligand of CYC and RAS1 receptors, was used as a reference for molecular docking^61^ and exhibited a binding energy of -7.0 kcal/mol toward both receptors. To identify ligands with stronger interactions and higher affinity for the targets, only compounds with lower binding energies than farnesol were considered^62^. The top two candidates were KCG, which showed a binding affinity of -9.4 Kcal/mol for the RAS1 receptor and − 10.3 kcal/mol for the CYC receptor, while IGR exhibited a binding affinity of -8.4 kcal/mol toward the RAS1 receptor and − 9.0 kcal/mol toward the CYC receptor (Supplementary Table 1). The docked complexes were visualized using Discovery Studio, and the interaction profiles of the four best-scoring complexes were presented in Supplementary Table 2. The 3D interactions between the lead compounds and the target proteins, highlighting key interactions, are depicted in Fig. 2.

### Free energy calculations

The Prime MMGBSA calculations for the six protein-ligand complexes indicate a wide range of binding affinities, highlighting significant differences in interaction stability. Among these, the RAS1-KCG complex stands out as the most stable, exhibiting the lowest binding free energy (ΔG = -127.125 kcal/mol), primarily driven by strong Coulombic (-51.887 kcal/mol) and Van der Waals (-59.750 kcal/mol) interactions. This indicates a highly favorable electrostatic and dispersion interaction profile. In contrast, the RAS1-farnesol complex displayed the weakest binding (ΔG = -49.478 kcal/mol), with significantly lower Coulombic (-6.597 kcal/mol) and Van der Waals (-24.524 kcal/mol) contributions, suggesting a less stable interaction. The CYC complexes exhibit intermediate binding energies, with CYC-farnesol showing a binding free energy of -66.004 kcal/mol and CYC-KCG demonstrating greater stability (-54.159 kcal/mol) due to enhanced Coulombic and Van der Waals interactions (Supplementary Table 3). These results underscore the importance of Coulombic and Van der Waals forces in shaping overall binding affinity.

### Physiochemical properties and drug-likeness

The chemical properties and drug-likeness profiles of the natural compounds were evaluated to assess their potential as QS inhibitor candidates. The results are summarized in Supplementary Table 4, which includes molecular weight, LogP values, number of H-bond donors and acceptors, topological polar surface area (TPSA), and compliance with Lipinski’s Rule of Five. Most compounds fulfill these criteria, although a few violated some rules. Nevertheless, these compounds may still exhibit significant biological activity, as is often the case with many natural bioactive molecules.

### Prediction of ADME-Tox properties

The in silico prediction of the ADME-Tox properties provides valuable insights into the safety profile of a compound^63,64^. As shown in Table 1, the KCG compound demonstrates moderate intestinal absorption (values below 30% indicate low absorption)^65^ and poor skin permeability (log Kp > -2.5 indicates low permeability)^66^. Its Caco-2 is also low, with a value below 0.90 (values > 0.90 suggest high Caco-2 permeability)^67^. Its blood-brain barrier (BBB) penetration is also low (compounds with a logBB > 0.3 are considered to cross the BBB readily, while those with a logBB < -1 are considered poorly distributed to the brain)^68^. KCG is neither a substrate nor an inhibitor of major CYP enzymes, such as CYP2D6 and CYP3A4, suggesting a low potential for drug-drug interactions. Additionally, its excretion profile shows a low clearance rate (a low total clearance value (log ml/min/kg) suggests that the drug has a long half-life), and it poses no significant risks in terms of genotoxicity, cardiotoxicity, or hepatotoxicity.

The IGR compound exhibits lower intestinal absorption and similarly poor skin permeability. Its ability to cross the BBB is even more restricted. Like the KCG compound, it is neither a substrate nor an inhibitor of CYP2D6 or CYP3A4 enzymes. Its toxicity profile is favorable, showing no evidence of AMES toxicity (a positive AMES test indicates potential mutagenicity of the compound^69^, hERG I inhibition, or hepatotoxicity with a low Caco-2 value. Thereby, both compounds appear to be promising candidates for MD simulations due to their strong binding affinities to the RAS1 and CYC proteins, as well as their toxicity profiles.

**Table 1:** In-silico prediction of ADME-Tox properties of the top two compounds (KCG and IGR).

	RAS1/CYC inhibitor	KCG	IGR
Absorption and distribution	Skin Permeability (log Kp)	-2.735	-2.735
	Intestinal absorption (human) (%)	64.302	21.706
	Caco2 permeability (log Papp in 10^− 6^ cm/s)	-0.034	0.273
	Blood-Brain Barier (Log BB)	-1.477	-1.997
Metabolism	CYP2D6/CYP3A4 substrates (Yes/No)	No	No
	CYP2D6/CYP3A4 inhibitors (Yes/No)	No	No
Excretion and toxicity	Total Clearance (log ml/min/kg)	-0.157	-0.164
	AMES toxicity	No	No
	Max. tolerated dose (human) (log mg/kg/day)	0.475	0.507
	hERG I inhibitor	No	No
	Hepatotoxicity	No	No

### MD simulations

MD simulations are a powerful computational method that provides dynamic insights into atomic-level processes within a given system^70^. These simulations enhance our understanding of the molecular environment and assist in the design of studies and targeted therapies. The selection of the two ligands, KCG and IGR, was based on their strong binding affinities, favorable interactions with the target proteins, and optimal ADMET profiles. In biomedicine, MD simulations are widely used to investigate how conformational changes in protein structures result from mutations or from ligand binding and unbinding^63^. This method provides valuable information about protein structures and protein-ligand interactions that are difficult to obtain using traditional techniques^64^. It offers a deeper comprehension of molecular mechanisms that are otherwise challenging to observe through conventional approaches^65^. MD analysis monitors both protein and ligand stability *via* RMSD, which measures the average displacement of selected atoms between a given frame and a reference frame, and RMSF, which characterizes local flexibility along the protein chain. RMSD and RMSF profiles highlight the dynamic behavior of the ligand within the protein’s active site^66^. Additionally, key interaction types, such as H-bonds and hydrophobic contacts, ionic interactions, and water bridges, are analyzed, providing insight into the strength and nature of ligand binding^67^. A timeline of these interactions is typically presented in two panels; the top panel shows the total number of specific contacts between the protein and ligand throughout the simulation trajectory. The bottom panel indicates which residues interact with the ligand at each trajectory frame. Residues forming multiple specific contacts are represented by darker shades of orange, according to the scale provided to the right of the plot. Ligand properties such as rGyr and SASA are also reported^68^.

#### MD analysis of CYC complexes

##### Analysis of RMSD

**Fig. 3:**
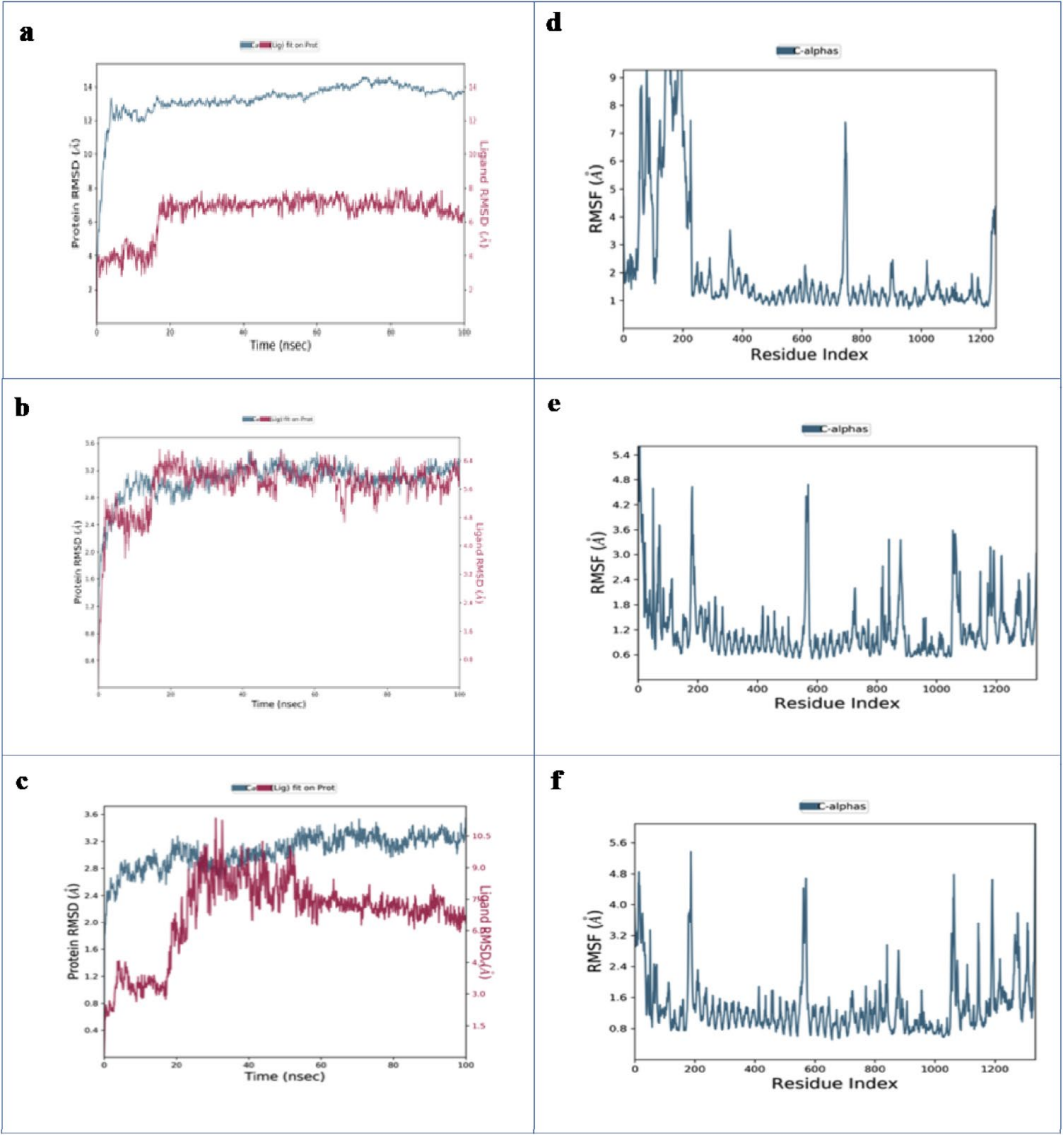
MD simulations analysis: (a) RMSD of CYC-farnesol complex; (b) RMSD of CYC-KCG complex; (c) RMSD of CYC-IGR complex; (d) RMSF of CYC-farnesol complex; (e) RMSF of CYC-KCG complex; (f) RMSF of CYC-IGR complex.

RMSD is used to calculate the average displacement of atoms in a specific frame relative to a reference frame^68,71^. It is computed for each frame along the simulation trajectory^72^ providing a measure of protein stability and structural changes over time^73^. In the RMSD plot of the CYC–farnesol complex over 100 ns (Fig. 3a), the protein backbone (Cα) shows an initial increase in RMSD within the first 10 ns, fluctuating between 10 and 14 Å. When the ligand is fitted to the protein, the RMSD first increases to approximately 5 Å and then stabilizes between 5 and 6 Å for the remainder of the simulation. The RMSD of the CYC-KCG complex was also monitored (Fig. 3b). During the first 10–15 ns, the protein exhibits notable fluctuations indicative of structural changes. Afterward, it stabilizes around 2.8 Å, with only minor deviations suggesting a stable conformation. In contrast, the ligand undergoes a significant increase in RMSD early in the simulation, reaching a peak near 6.2 Å, which suggests substantial conformational changes. Then it stabilizes between 5.8 and 6.0 Å after 15–20 ns. For the CYC-IGR complex (Fig. 3c), the protein’s RMSD gradually increases over the first 20 ns before stabilizing around 3.2 Å with minimal fluctuations for the remainder of the simulation, indicating a relatively stable conformation. Conversely, the ligand initially exhibits lower RMSD values, but after 25 ns, the RMSD increases significantly, peaking at 9 Å before gradually stabilizing at 7 Å. These higher fluctuations are likely due to conformational changes or movements within the binding site before the ligand reaches a more stable configuration, particularly between 20 and 60 ns.

Compared to farnesol, the ligands KCG and IGR display distinct stability and conformational dynamics. The CYC-KCG complex stabilizes at a lower RMSD (2.8 Å), whereas the CYC-farnesol complex fluctuates between 10 and 14 Å. However, the ligand RMSD for KCG indicates moderate conformational changes during the early stages of the simulation, stabilizing between 5.8 and 6.0 Å. In contrast, IGR exhibits higher ligand fluctuations, peaking at 9 Å and stabilizing at 7 Å, indicating significant conformational changes within the binding site. These results indicate that KCG leads to greater protein stability compared to both IGR and farnesol.

##### Analysis of RMSF

The RMSF plot shows the flexibility of residues within a protein structure, with the residue index on the X-axis and RMSF values in Ångströms on the Y-axis^74^. Lower RMSF values indicate reduced atomic fluctuations, suggesting greater structural stability in those regions^75^. Figure 3d presents the RMSF plot of the CYC-farnesol complex, highlighting regions of residue flexibility. Notable peaks are observed, particularly between residues 50–200 and near residue 800, indicating increased flexibility in these regions. For the CYC-KCG complex (Fig. 3e), several peaks are observed, mainly between residues 0-200 and 600–1000, with fluctuations reaching up to 4.8 Å. However, most residues between 200 and 600 exhibit low fluctuations, generally below 2 Å, indicating relative structural stability. In contrast, the CYC-IGR complex (Fig. 3f), shows fluctuation peaks reaching approximately 5.6 Å, particularly around residues 200, 600, and 1000. The majority of residues display moderate fluctuations, with RMSF values ranging between 2 and 3.5 Å.

Based on the RMSF analysis, the CYC-KCG complex demonstrates the greatest structural stability, with most residues between 200 and 600 fluctuating below 2 Å. The CYC-farnesol complex shows moderate stability, although the high RMSF peaks indicate lower conformational stability. CYC-IGR complex exhibits the highest fluctuation peaks (up to 5.6 Å) around residues 200, 600, and 1000, suggesting a less stable interaction. Overall, KCG emerges as the most promising ligand due to its superior stability profile in complex with the CYC protein.

##### Interaction analysis and contacts timeline

**Fig. 4:**
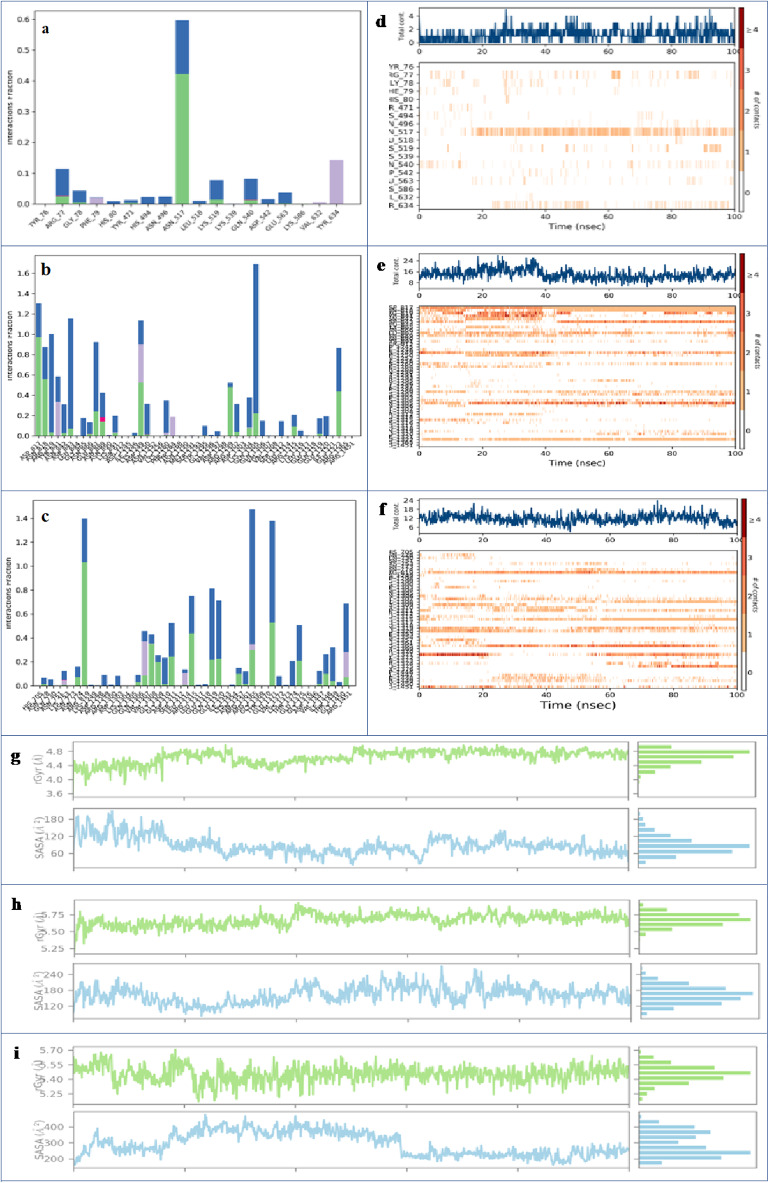
MD simulations analysis. (a) Histogram of CYC-farnesol interactions; (b) Histogram of CYC-KCG interactions; (c) Histogram of CYC-IGR interactions; (d) CYC-farnesol timeline; (e) CYC-KCG contact timeline; (f) CYC-IGR contact timeline; (g) rGyr and SASA of farnesol; (h) rGyr and SASA of KCG; (i) rGyr and SASA of IGR in complex with CYC during 100 ns MD simulation.

Protein interactions with ligands can be observed and categorized by type while the simulation is running^76^. The plot below summarizes these interactions, commonly referred to as contacts, which are typically classified into four main categories (ionic interactions, water bridges, hydrophobic interactions, and H-bonds)^77^. The Fig. 4a illustrates the main interactions that occurred between CYC and farnesol during the simulation. ASN496 shows the highest interaction percentage (0.6), mainly due to water bridges and H-bonds, indicating its critical role in stabilizing the ligand. While ARG77 occasionally forms H-bonds, TYR76 contributes moderately through water bridges and H-bonds. GLY78, PHE79, and HIS80 exhibit only minor contributions, while HIS494 and TYR471 are involved in water-mediated interactions. Notably, TYR634 and VAL632 enhance ligand stability through hydrophobic contacts. Receptor-ligand contact timelines are shown in Fig. 4d in two panels. The top panel shows a consistent variation between 2 and 4 contacts over time, suggesting a stable yet dynamic binding interaction. Some residues, such as ASN496 and LYS519, maintain persistent and frequent contacts beyond 10 ns.

For the KCG ligand (Fig. 4b), significant interactions were observed with several amino acids. ASP817, with a fraction of 1.3, showed H-bonds and water bridges. ASN818 had a fraction of 0.9, while ARG819 presented a fraction of 1.0, both characterized by H-bonds and water bridges. Additionally, LYS1249 formed three types of interactions (H-bonds, water bridges, and hydrophobic contacts) with a fraction of 1.2. GLN1305 reached a fraction of 1.6, while GLU1322 showed a fraction of 0.8; both residues engaged in H-bonds and water bridge interactions. The total number of contacts ranged between 8 and 24 and stabilized after 40 ns. The amino acids ASP817, ASN818, ARG819, LYS1249, and GLN1305 maintained frequent contacts (8–16) with strong interaction intensity represented in orange throughout the simulation (Fig. 4e). The ligand IGR exhibits several notable interactions (Fig. 4c ). ASN819, with a fraction of 1.4, forms H-bond as well as water bridges interactions. Similarly, ARG1315, GLN1319, and GLU1320, each with a fraction of 0.8, display similar interactions involving H-bonds and water bridges. Furthermore, ARG1365, with a fraction of 1.4, shows not only H-bonds and water bridges but also hydrophobic interactions. TYR1370, with a fraction of 1.3, also contributes through H-bonds and water bridges. The total number of contacts ranges between 6 and 18, with noticeable stabilization after 20 ns. The residues ASN819, ARG1315, GLN1319, GLU1320, and TYR1370 maintain consistent contacts (8–16) during the simulation, with strong interaction intensities represented in orange (Fig. 4f ).

Among the three ligands, KCG exhibits the most robust and stable binding, supported by a diversity of interactions, including H-bonds, water bridges, and hydrophobic contact. Farnesol shows fewer interactions, while IGR exhibits moderate contacts. Overall, KCG emerges as the most effective ligand in terms of interaction strength and stability.

##### Ligands properties

The plots of the Fig. 4 present the rGyr and SASA of farnesol (Fig. 4g), KCG (Fig. 4h), and IGR (Fig. 4i). In the upper panel, the rGyr of farnesol remains relatively constant between 4.2 and 4.6 Å, indicating consistent compactness throughout the simulation. The lower panel shows moderate SASA fluctuations, ranging approximately from 60 to 160 Å², suggesting variable surface exposure. KCG maintains a stable rGyr (5.5–5.7 Å), but its SASA varies more significantly, fluctuating between 120 and 240 Å². This indicates a flexible structure with variable interactions with the solvent. IGR displays a more variable rGyr (5.3–5.6 Å), depicting a compact yet slightly dynamic structure. IGR’s SASA exhibits the widest range (~ 200–400 Å²), suggesting greater solvent exposure and higher structural flexibility.

##### Interactions: hydrogen bonding and water

**Fig. 5:**
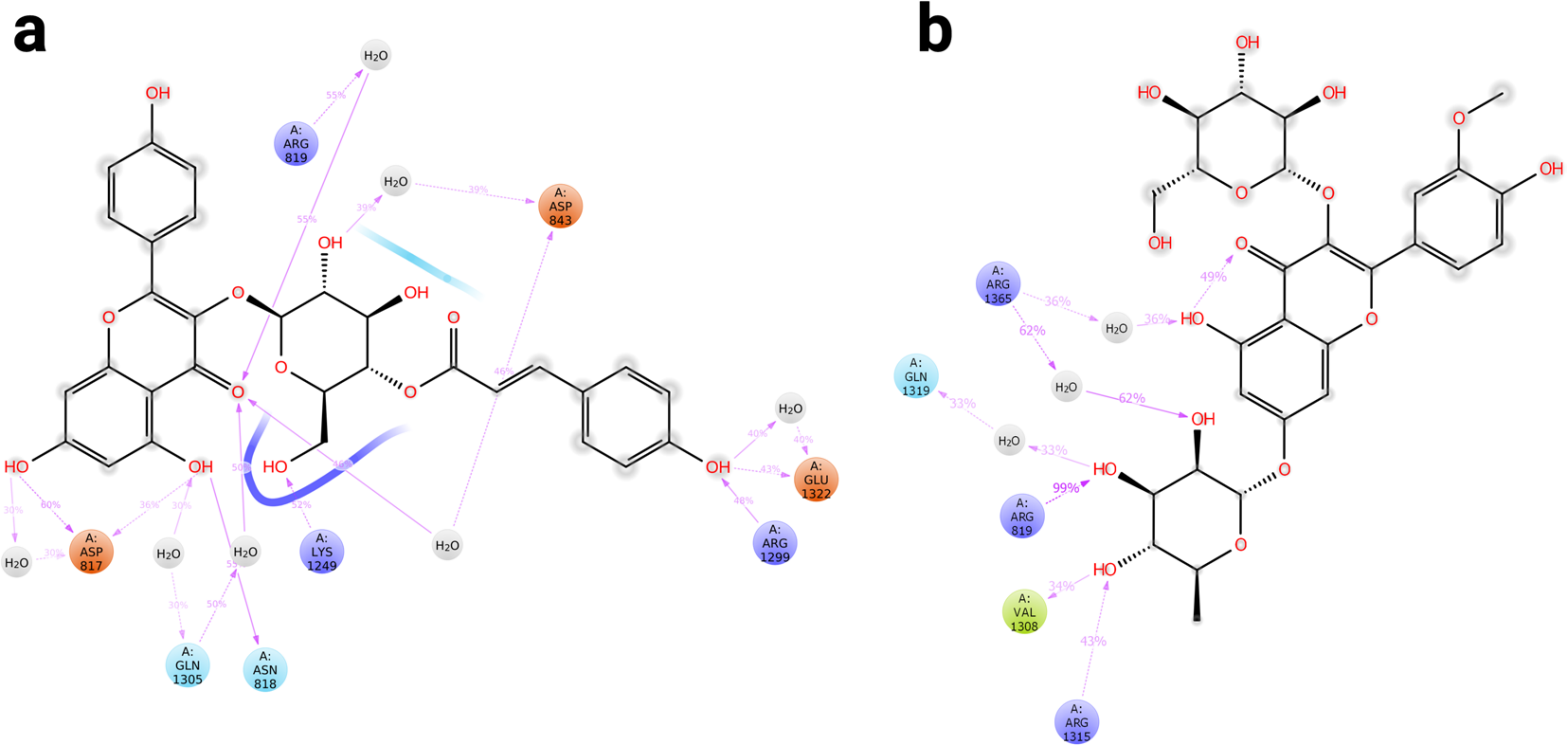
2D Diagram of Hydrogen Bonding Network and Water-Mediated Interactions. (a) KCG-CYC receptor and (b) IGR-CYC receptor.

Figure 5a illustrates the interactions between KCG and several amino acid residues within the CYC protein. Among these interactions, ARG819 forms an H-bond with a water molecule at 55%, while ASP843 and ASP817 interact with water molecules at 39% and 30% respectively. GLN1305 and ASN818 form similar water bridge interactions with frequencies of 50% and 55%, respectively. In the central region, H-bonds are observed between the hydroxyl group of the ligand and LYS1249 (52%), along with water-mediated interactions with GLU1322 (43%) and ARG1299 (48%).

The Fig. 5b shows the interactions of IGR with several amino acid residues of the CYC receptor through H-bonds and water-mediated contacts. The ligand, which contains hydroxyl (-OH) and carboxyl (-COOH) functional groups, interacts with residues such as ARG1365, GLN1319, ARG819, VAL 1308, and ARG1315. ARG1365 forms water-mediated interactions with frequencies of 36% and 62%, while GLN1319 interacts *via* water bridges at 33%. ARG819 displays a highly stable H-bond with the hydroxyl group at 99%. VAL1308 and ARG1315 show interaction frequencies of 34% and 43%, respectively.

#### Analysis of RAS1 complexes

##### Analysis of RMSD

**Fig. 6:**
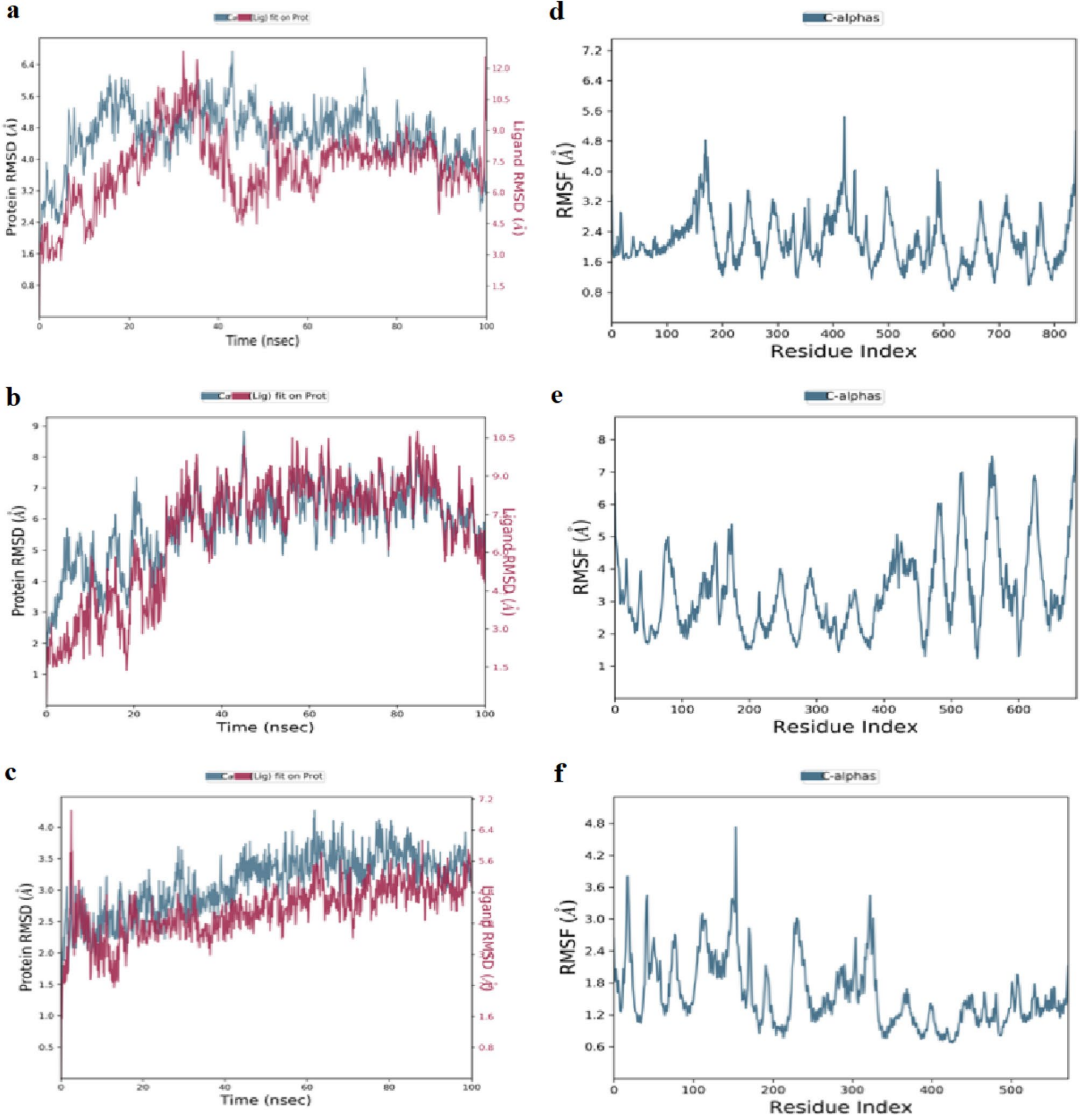
MD simulations analysis: (a) RMSD of RAS1-farnesol complex; (b) RMSD of RAS1-KCG complex; (c) RMSD of RAS1-IGR complex; (d) RMSF of RAS1-farnesol complex; (e) RMSF of RAS1-KCG complex; (f) RMSF of RAS1-IGR complex.

Both protein and ligand RMSD curves increase in the RAS1-farnesol plot (Fig. 6a). After 30–40 ns, the RMSD of the RAS1 protein stabilizes between 3.5 and 6.0 Å, indicating that it reaches a relatively stable state with only slight variations. Additionally, the ligand RMSD also stabilizes, fluctuating between 2.5 and 5.0 Å. After around 40 ns, both protein and ligand trajectories exhibit overall stability. In the RAS1-KCG complex (Fig. 6b), the protein’s RMSD increases significantly within the first 20 ns, reaching approximately 7.5 Å. It then stabilizes, fluctuating between 6 and 8 Å. Simultaneously, the ligand RMSD begins at 1.5 Å, indicating a stable initial binding pose, but then rises drastically, peaking at approximately 9.5 Å by 40 ns. Remarkably, the ligand’s RMSD decreases after 60 ns. The patterns point to a dynamic interaction between the ligand and protein, where the ligand’s mobility and binding stability are influenced by the protein’s structural rearrangements. This underscores the adaptive and flexible nature of the ligand-protein complex^78^. The Fig. 6c illustrates the RMSD evolution for the RAS1-IGR complex. The protein RMSD starts below 2 Å, gradually increases, peaking at 3.5 Å by 50 ns, and then fluctuates between 3.5 and 4 Å for the remainder of the simulation. The ligand RMSD begins around 2 Å, fluctuates slightly, peaking at 6.5 Å, and then stabilizes between 4.5 and 6 Å for most of the trajectory. To sum up, among the two ligands, KCG demonstrates superior dynamic behavior and structural adaptability when interacting with the RAS1 protein, making it the most promising candidate in terms of flexibility and stability.

##### Analysis of RMSF

The RMSF plot in (Fig. 6d) highlights the dynamic behavior of the RAS1-farnesol complex across its residue sequence during the simulation. Residues 100–180 exhibit low RMSF values (0.8–2.4 Å). In contrast, residues around position 400 show high RMSF peaks (4–7 Å). The RMSF profile of the RAS1-KCG complex (Fig. 6e), reveals that the fluctuations are generally below 4 Å for the majority of residues. However, occasional peaks exceeding 6 Å are observed, particularly between residues 500 and 650. The RMSF profile of the RAS1-IGR complex (Fig. 6f) indicates that residues near indices 100 and 200 display higher fluctuations, with RMSF values exceeding 3.5 Å. Whereas, residues ranging from 300 to 450 exhibit minor fluctuations, typically ranging from 1 to 2 Å. The RMSF analysis reveals distinct dynamic behaviors among the three RAS1 complexes. The RAS1-farnesol complex shows very high flexibility, especially around residue 400, where RMSF values range from 4 to 7 Å, while residues 100–180 remain relatively stable with lower RMSF values (0.8–2.4 Å). The RAS1-KCG complex displays overall greater stability, with most residues fluctuating below 4 Å. However, around residue indices 500 and 650, RMSF values exceed 6 Å. The RAS1-IGR complex exhibits higher fluctuations at residues 100 and 200 (RMSF > 3.5 Å), whereas, the region between 300 and 450 remains stable (1–2 Å). This suggests that KCG provides the most stabilizing effect on the RAS1 protein, while farnesol shows flexibility in specific areas, and IGR presents a mixed dynamic profile with both stable and flexible regions.

##### Interaction analysis and contacts timeline

**Fig. 7:**
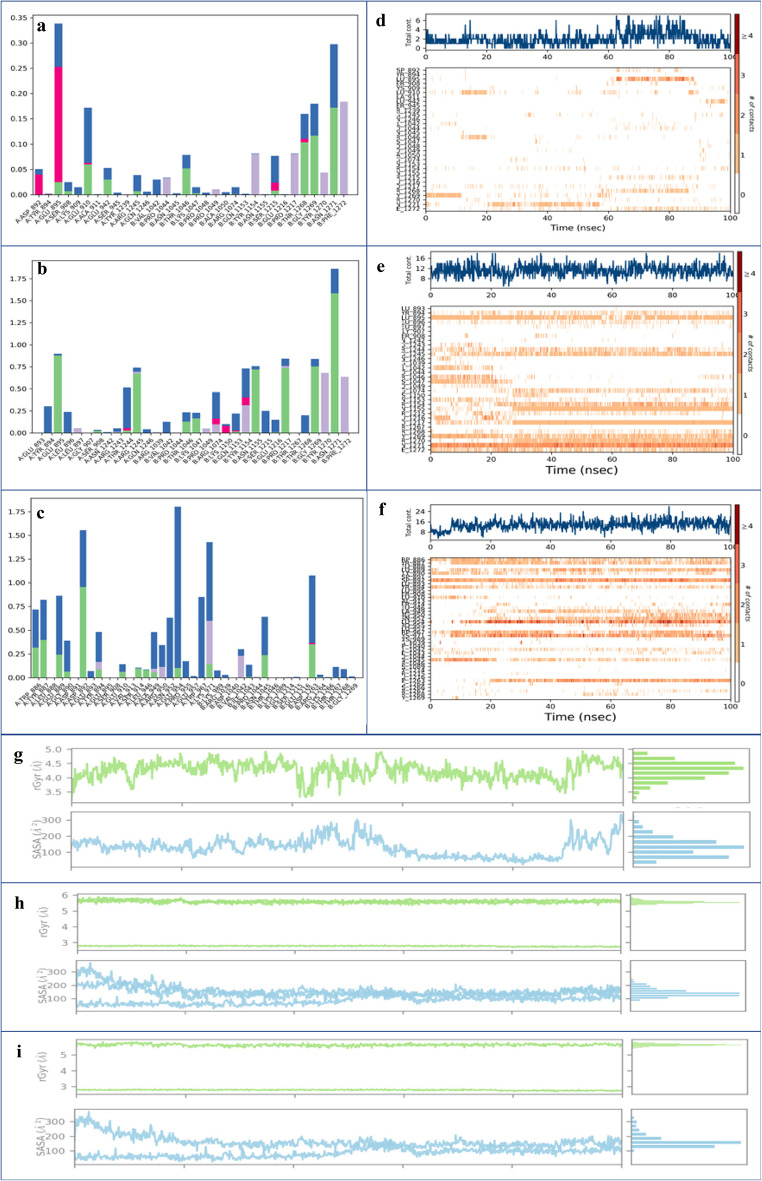
MD simulations analysis, interactions, and contacts timeline plots. (a) Histogram of RAS1-farnesol interactions; (b) Histogram of RAS1-KCG interactions; (c) Histogram of RAS1-IGR interactions; (d) RAS1-farnesol contact timeline; (e) RAS1-KCG contact timeline; (f) RAS1-IGR contact timeline; (g) rGyr and SASA of farnesol; (h) rGyr and SASA of KCG; (i) rGyr and SASA of IGR in complex with RAS1 during 100 ns MD simulation.

Several types of interactions between RAS1 and farnesol during the simulation are observed (Fig. 7a). The most significant interactions occur with residues ASN1271, PHE1272, and GLU895, all of which exhibit high interaction fractions. Notably, GLU895 engages both ionic and water bridge interactions, suggesting a crucial role in stabilizing the ligand. ASN1271 forms water bridge and H-bond interactions, while PHE1272 displayed hydrophobic contacts. The Fig. 7d presents the interaction timeline, showing the number of contacts between the protein and the ligand at each time point. Although the number of interactions fluctuates, it typically remains between two and six contacts throughout the simulation. Darker orange shades indicate a higher number of contacts. Key residues exhibiting frequent and consistent interactions include GLU895, THR1268, ASN1271, and PHE1272. Figure 7b and e illustrate the correlation between residue contacts and time evolution for the RAS1-KCG complex. Significant interactions were observed with various amino acids, including TRP886 (fraction 0.75), TYR887 (fraction 0.90), and ASP892 (fraction 1.50). GLU954 reached a fraction of 1.75, and ASN1045 (fraction 0.75) was characterized by H-bonds and water bridge interactions. TRP967 (fraction 0.95) displayed water bridge interactions, while LYS971 (fraction 1.50) exhibited H-bonds, water bridges, and hydrophobic interactions. ASP1263 (fraction 1.25) showed ionic, water bridge, and H-bond interactions. After 20 ns, these interactions became apparent, with a total of 16 contacts. These amino acids demonstrated consistent interactions throughout the simulation. The Fig. 7c shows the residue interactions of the RAS1-IGR complex. GLU895 (0.90) forms H-bonds and water bridges, while THR1244 (0.50) shows ionic, H-bond, and water bridge interactions. ARG1245 (0.75) and ASN1155 (0.75) both exhibit H-bonds and water bridges interactions. TYR1154 (0.75) displays hydrophobic, water bridge, and ionic interactions. PRO1217 (0.80), GLY1269 (1.0), and ASN1271 (1.75) form H-bonds and water bridges, whereas PHE1272 (0.60) shows only hydrophobic interactions. These contacts persisted from 20 ns onward, showing a total number of contacts between 8 and 12 (Fig. 7f). Additionally, these amino acids exhibited frequent contacts throughout the simulation.

The simulation results highlight distinct but significant interaction profiles between RAS1 and the three ligands. Farnesol primarily interacts with residues such as GLU895, ASN1271, and PHE1272 through a combination of ionic, water bridge, hydrophobic, and H-bond contacts, which contribute to ligand stabilization and binding. KCG exhibits strong and consistent binding, particularly with residues TRP886, TYR887, ASP892, GLU954, and LYS971, through H-bonds, water bridges, and ionic interactions. These interactions play a critical role in maintaining the stability of the complex. The main binding residues for IGR include GLU895, THR1244, ARG1245, ASN1155, and PHE1272, through a combination of hydrophobic, H-bonds, and ionic interactions, supported by water bridges contacts. In the performed simulations, all three ligands exhibit consistent and stable interactions throughout the simulation, contributing to high binding affinity and stability within the RAS1 protein. However, as detailed above, notable differences exist in the types of interactions and the specific residues involved for each ligand.

##### Ligands properties

The rGyr and SASA plots reflect the behavior of farnesol (Fig. 7g), KCG (Fig. 7h), and IGR (Fig. 7i) when bound to RAS1 protein during MD simulations. For farnesol, rGyr fluctuates between 4.0 and 4.8 Å, showing moderate structural flexibility, while SASA values vary significantly (~ 100–300 Å^2^, indicating dynamic surface exposure and conformational changes throughout the simulation. The KCG-RAS1 complex exhibits the highest structural stability with minimal variations in rGyr (~ 5.5 Å), and a relatively consistent SASA between ~ 150 and 250 Å^2^ suggesting a compact conformation with limited solvent exposure. Similarly, the IGR-RAS1 complex demonstrates a stable rGyr around 5.4 Å, with slight fluctuations. It’s SASA values range from 150 to 250 Å^2^ indicating intermediate solvent exposure and moderate structural rigidity.

##### Interactions: hydrogen bonding and water

**Fig. 8:**
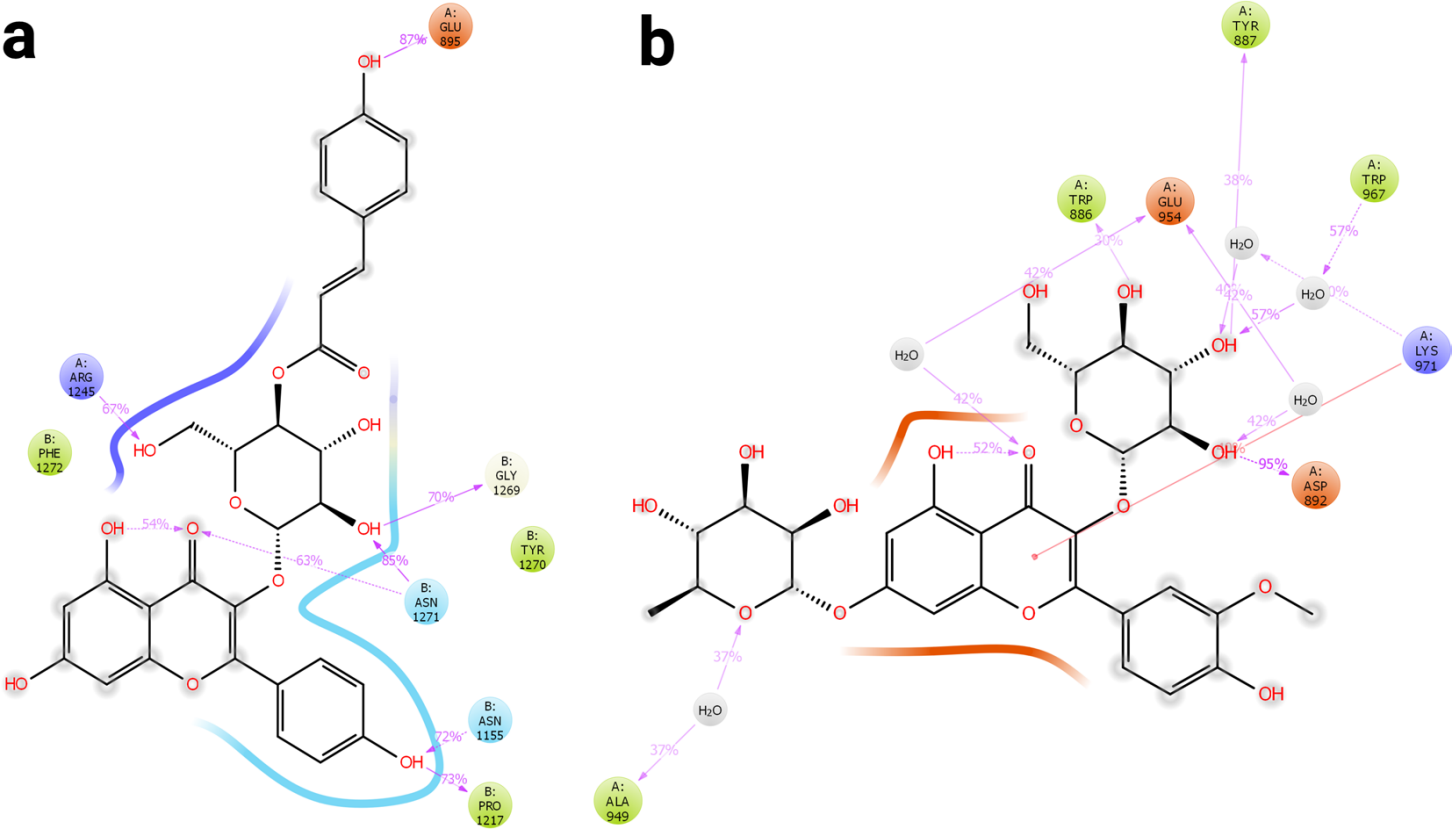
2D diagram of hydrogen bonding network and water-mediated interactions. (a) KCG-RAS1 receptor and (b) IGR-RAS1 receptor.

The Fig. 8a illustrates various interactions between the KCG and surrounding amino acids, with percentages indicating the frequency over the course of the simulation. GLU895 forms an H-bond (with a frequency of 87%), PRO1217 and ARG1245 exhibits both H-bond and water bridge interactions with frequencies of 73% and 67%, respectively. PHE1272 is likely involved in hydrophobic interactions with the ligand’s non-polar regions. GLY1269 also shows a notable interaction frequency (70%). ASN1271 (63%) and ASN1155 (72%) residues are involved in polar interactions, most likely via H-bonding. The Fig. 8b presents the interactions between the IGR ligand and its surrounding amino acids. Notable residues include ASP892 with a high interaction frequency (95%), which likely forms a strong H-bond. GLU954 also contributes significantly through hydrogen bonding (42% interaction frequency). Other residues such as TRP886 (30%) and TRP887 (38%) are likely involved in hydrophobic interactions, further stabilizing the complex.

The MD simulations reveal distinct stability and interaction profiles for the complexes formed by farnesol, KCG, and IGR with the targeted druggable receptors. Farnesol exhibits relatively stable interactions, with key residues such as ASN496 and GLU895 contributing to its stabilization through H-bonds and hydrophobic interactions. The RMSD also stabilizes after initial fluctuations. Comparatively, KCG and IGR display more complex interaction profiles, with KCG demonstrating stronger and more persistent interactions, especially with residues like LYS1249 and GLN1305. In contrast, IGR shows higher RMSD peaks and forms notable interactions with residues such as ARG1365 and ASN819, supported by H-bonds and water bridges. Overall, KCG demonstrates superior and robust interaction profiles, making it a promising candidate. Although IGR shows moderate stability, it can still be considered as a potential candidate. Both KCG and IGR exhibit favorable binding characteristics that warrant further interest.

## Superimposition analysis of proteins and protein-ligand complexes

**Fig. 9:**
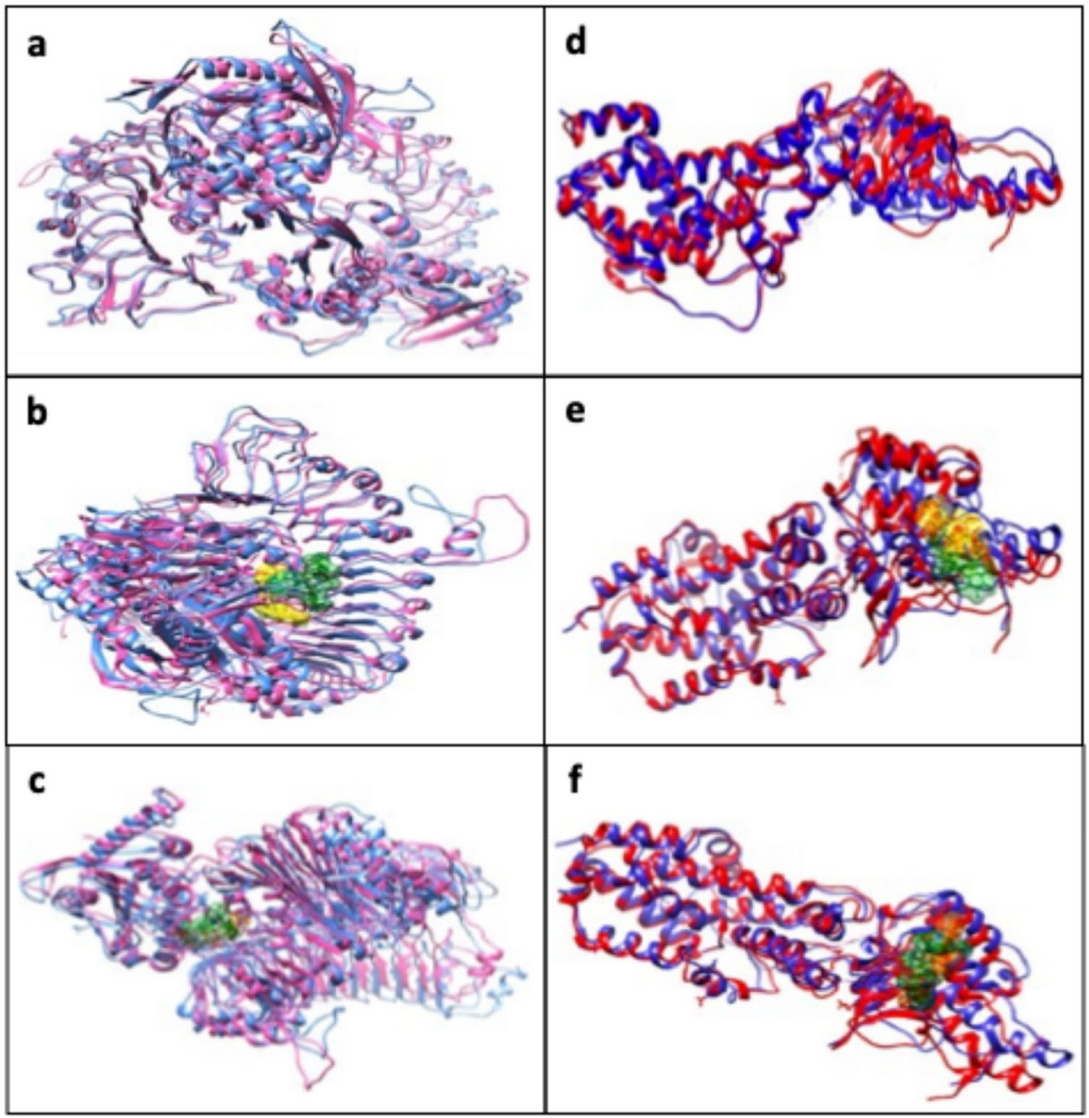
Superimposed 3D structures of proteins and protein-ligand complexes at the initial (0 ns) and final (100 ns) frames of molecular dynamics simulation. (a) CYC protein 3D superposition; (b) CYC-IGR 3D superposition; (c) CYC-KCG 3D superposition; (d) RAS1 protein 3D superposition; (e) RAS1-IGR 3D superposition; (f) RAS1-KCG 3D superposition.

To assess the structural stability of proteins and their complexes during MD simulations, we performed a 3D superposition between representative structures from 0 ns (initial frame) and 100 ns (final frame). As shown in the Fig. 9, the conformations of both CYC and RAS1 proteins remained generally stable, with only slight deviations observed in flexible regions.

For CYC (Fig. 9a), the hot pink (0 ns) and cornflower blue (100 ns) colored structures show near-perfect alignment, indicating minor conformational variation. When complexed with IGR (Fig. 9b) and KCG (Fig. 9c), these ligands remain firmly anchored in the binding site, with limited displacements reflecting minor conformational adjustments, with RMSD values of 1.237 Å and 1.247 Å, respectively. Similarly, the RAS1 protein (Fig. 9d) displayed structural consistency between 0 ns (blue) and 100 ns (red), indicating overall conformational stability. In the RAS1-IGR (Fig. 9e) and RAS1-KCG (Fig. 9f) complexes, the ligands remain embedded within the binding site, with no signs of dissociation or drastic displacement, with also low RMSD values (1.121 Å and 1.267 Å, respectively), indicating excellent stability.

The 3D superposition analyses performed between the initial (0 ns) and final (100 ns) frames revealed a high structural stability of CYC and RAS1 proteins, both in the free state and when bound to IGR and KCG ligands. The low RMSD values, all below 1.3 Å, confirm the overall stability. In addition, the absence of significant displacement of the ligands within the binding pocket suggests a stable and strong interaction between the candidate molecules and their respective targets. These observations indicate a promising inhibitory potential for KCG and IGR compounds, which fully justifies their selection for further investigations.

## Discussion

Propolis has demonstrated significant antimicrobial activity against food pathogens and toxigenic fungi^79^. However, the precise mechanisms underlying its action remain largely unclear. Although its efficacy is well established, propolis is often tested as a complex mixture, making it difficult to precisely identify its active components and their mechanisms of action. Moreover, there is a lack of focused work on understanding its effects at the molecular level and how it might specifically interfere with complex biological systems like QS^80,81^.

SBDD approach offers the advantage of enabling detailed analysis of interactions between ligands and molecular targets. This method identifies therapeutic candidates by using computer models and simulations to predict interactions at the atomic level^82^ underscoring the significance of SBDD in novel drug discovery^83^. Furthermore, drugs developed through CADD, such as the hepatitis C protease inhibitor boceprevir and certain antiviral agents targeting human immunodeficiency viruses (HIV), have corroborated the effectiveness of this approach in identifying molecules with specific therapeutic properties^84,85^.

In this study, we combined molecular docking and MD simulations to investigate the potential of Moroccan propolis-derived molecules as inhibitors of the QS receptors, CYC and RAS1, in *C. albicans*. Since QS plays a pivotal role in regulating the pathogenicity of *C.albicans*, targeting these proteins represents a promising therapeutic strategy^86,87^. Farnesol is highly effective at inhibiting biofilm formation and can also disrupt pre-formed biofilms^88^. When comparing the reference ligand farnesol to KCG and IGR ligands, notable differences in binding affinities and interaction profiles emerge. Farnesol demonstrated good interactions with the target proteins, as evidenced by a binding affinity of -7.0 kcal/mol. IGR and KCG compounds showed stronger binding affinities, suggesting that they may serve as more effective ligands. Previous studies support this, indicating that flavonoids, such as kaempferol derivatives, exhibit significant biological activity due to their strong binding and persistence in protein active sites^89^. In terms of interaction mechanism, farnesol primarily interacts with proteins through hydrophobic contacts. While these contacts support binding, they do not provide the same level of stability as more diverse interactions. In contrast, IGR and KCG display a broader range of interactions, which contribute to greater binding specificity and complex stability. These interactions are critical in maintaining a strong, lasting bond with the protein and enhancing inhibitory activity^90^. Taken together, although farnesol demonstrates some inhibitory potential, the higher binding affinities and robust interaction profiles of KCG and IGR suggest that they are more promising candidates for further exploration as therapeutic agents.

According to Prime MMGBSA binding free energy calculations, the variation in stability among the protein-ligand complexes was highly significant. The superior binding affinity of the RAS1–KCG complex was primarily driven by stronger Van der Waals and Coulombic interactions, making it a stable and energetically favorable complex. Strong binding is also very much indicative of excellent electrostatic complementarity and extensive hydrophobic contacts between KCG and the RAS1 protein, further supporting its potential as a promising inhibitor. In contrast, RAS1-farnesol exhibited the weakest binding contributions, which were substantially lower, suggesting a less stable interaction. Among the CYC complexes, CYC-KCG showed stronger binding than CYC-farnesol, likely due to more favorable electrostatic and hydrophobic interactions. These findings collectively underscore the pivotal role of coulombic and Van der Waals forces in determining the overall binding strength and complex stability.

In the MD simulations, RMSD and RMSF were calculated to monitor the overall stability of the protein-ligand complex and the local flexibility of residues, respectively. The number and types of intermolecular interactions (H-bonds, hydrophobic contacts, ionic interactions, and water bridges) were tracked throughout the simulation trajectory. Interaction timelines were generated to visualize how each residue made contact with the ligand during the course of all frames. Also, rGyr and SASA were evaluated to assess ligand compactness and solvent exposure, respectively^91^. These combined parameters provided detailed insights concerning binding stability, flexibility, and interaction dynamics within protein-ligand systems. The interactions of the two ligands, KCG and IGR, were analyzed in relation to the two druggable receptors, CYC and RAS1. When analyzing the KCG ligand with CYC, the RMSD showed initial fluctuations, but the complex stabilized after 15–20 ns, with the ligand’s RMSD reaching approximately 5.8-6.0 Å. KCG formed significant interactions with residues such as ASP817, ARG819, and LYS1249 through H-bonds, water bridges, and hydrophobic interactions, all contributing to the complex’s overall stability. In contrast, the IGR-CYC complex displayed larger fluctuations, with the ligand RMSD peaking at 9 Å before stabilizing at 7 Å later in the simulation. While residues such as ARG1365 and ASN819 contributed to the stabilization of IGR through H-bonds and water bridges, the higher RMSD values indicate higher conformational changes compared to KCG.

For RAS1 receptor, KCG molecule also showed favorable results, stabilizing after 20 ns with RMSD values ranging between 6 and 8 Å. Strong interactions with residues like ASP892 and LYS971, involving H-bonds and water bridges, contributed to the stability of this complex. IGR, when bound to RAS1, showed higher fluctuations, with RMSD values peaking around 6.5 Å; however, the complex remained relatively stable due to interactions with residues GLY1269 and ARG1245 through H-bonds. The RMSD analysis showed that KCG has a more stable protein conformation compared to both farnesol and IGR. While farnesol demonstrated significant stability, IGR showed higher fluctuations. These findings were further supported by RMSF analysis showing that IGR experienced larger fluctuations than both KCG and farnesol. Thus, KCG and farnesol demonstrated superior stability.

Comparing the two ligands, KCG and IGR, KCG appears to be the better candidate overall due to its consistently lower RMSD values, indicating greater stability in both protein systems (CYC and RAS1), and its ability to form more robust and diverse interactions, especially with CYC, where it demonstrated strong H-bonding and water-mediated interactions. When comparing our findings with existing studies, the observed interactions between KCG and the protein targets align closely with the known bioactivity and binding affinity of this flavonoid, considering that KCG is a derivative of kaempferol. Several studies have demonstrated that kaempferol derivatives exhibit potent protein binding due to their ability to form H-bonds and hydrophobic interactions^92,93^. The literature also emphasizes that kaempferol is known for its ability to disrupt protein-protein interactions in cancer pathways, supporting its potential for therapeutic applications^94,95^. Zhou et al., highlighted kaempferol’s superior pharmacological profile due to its robust interactions within biological systems^96^. Whereas, Gong et al., reported that isorhamnetin often shows lower bioactivity and binding affinity compared to other flavonoids^97^.

Our observations align with the increasing number of reports highlighting the diverse bioactivities of propolis beyond its traditional antimicrobial effects. For example, a recent study by Cora et al.,^98^ reported that Turkish propolis possesses strong antioxidant, antimicrobial, antiviral, and antiproliferative activities due to its high content of phenolics and flavonoids. Notably, the inhibition of QS activity was also reported in their study; however, no anti-swarming or antibiofilm activities were observed. Čengić et al.^99^ also characterized the antimicrobial potency of chestnut honey, pollen, and propolis against various pathogens, including *C. albicans*. Interestingly, a 20% concentration of propolis extract mixed with chestnut honey and pollen (apimixture A3) almost completely inhibited all tested microorganisms, including *C. albicans*. These findings support our in silico study identifying propolis-derived compounds as potential QS inhibitors in *C. albicans*. The demonstrated antimicrobial synergy within the apimixtures suggests that propolis constituents may interfere with QS pathways to reduce virulence. This reinforces our approach of mitigating pathogenicity by targeting the QS mechanism. Moreover, the study highlights the potential of natural product mixtures, implying that the use of propolis in conjunction with other bee products could contribute towards a more potent therapeutic efficacy.

The study conducted by Hadjab and his team^100^ concentrated on the biological activity and chemical profile of two ethanolic extracts of Algerian propolis, specifically their antimicrobial, antioxidant, antibiofilm, and anti-quorum sensing properties. The propolis extract from the Guelma region exhibited the highest levels of flavonoids and total phenolics, with cynarin as the major compound identified. The extract was considerably effective against multidrug-resistant (MDR) strains, by inhibiting biofilm formation. Finally, moderate QS inhibition was evidenced by a concentration-dependent reduction in violacein production in *Chromobacterium violaceum* ATCC 12,472.

Kolaylı et al.^101^ conducted a study on four ethanolic propolis extracts, analyzing their phenolic compositions and evaluating their antimicrobial, antioxidant, anti-quorum sensing, and anti-biofilm activities. Depending on their composition, the extracts exhibited varying degrees of antimicrobial activity against pathogens such as *S. aureus*, *C. violaceum*, *M. smegmatis*, *B. cereus*, *C. albicans*, and *C. parapsilosis*. All extracts were particularly effective in anti-QS and anti-biofilm activities against *Chromobacterium violaceum* and *Pseudomonas aeruginosa*, with higher phenolic content correlating with greater bioactivity. These findings support the notion that certain natural compounds in propolis can interfere with bacterial and fungal communication, thus supporting our in silico identification of propolis-derived QS inhibitors targeting *C. albicans*.

A limitation of our study is its exclusive reliance on computational methods. We did not evaluate the anti-QS and antifungal activities of KCG and IGR through in vitro or in vivo experiments. Further research is necessary to validate our findings. Despite minor deviations from Lipinski’s Rule of Five, which predicts oral bioavailability, KCG and IGR demonstrated strong binding affinities, and the extent of these violations remains limited. Nevertheless, recent studies suggest that many natural compounds with therapeutic potential can be optimized using advanced drug delivery systems. For example, lipid encapsulation, nanoparticles, polymeric carriers, and nanomicelles can enhance bioavailability by overcoming solubility or permeability issues^102,103^. Pirie et al. claim that effective oral drug development can extend beyond the traditional thresholds set by the “Rule of Five”^104^. Similarly, Ahmed et al.^105^ contend that, even if hesperidin and naringin do not fully comply with Lipinski’s rule, their therapeutic potential warrants further investigation into their bioavailability and in vivo efficacy. Ganesan^106^ also emphasizes that, although some natural products do not satisfy most of the traditional drug-like criteria, they remain valuable scaffolds in drug discovery due to their ability to interact with biological targets.

Globally, researchers have increasingly focused on QS-mediated infectious diseases. Attention has shifted toward QS inhibitors as antipathogens, to address the persistent failure of antibiotics in combating microbial infections^107^. In this context, farnesol was initially discovered 22 years ago as a QS molecule that plays a pivotal role in inhibiting the yeast-to-hyphae transition in *C. albicans*^108^. However, its role in *Candida* spp. biology is unexpectedly complex^109^. When secreted, farnesol, can also act as a virulence factor during infection and as a fungicide by inducing apoptosis in competing fungi^110^. The KCG compound may serve as a viable therapeutic option for *C. albicans* infections by inhibiting QS, potentially reducing pathogenicity without promoting resistance.

## Conclusion

This study presents an in silico screening of 106 compounds derived from Moroccan propolis, identifying KCG as a promising lead candidate for QS inhibition in *C. albicans*. KCG exhibits strong binding affinities and forms stable complexes with the key QS regulators, CYC and RAS1, as evidenced by molecular docking and MD simulations. Moreover, KCG’s favorable ADMET profile supports its potential applicability in targeting QS pathways, thereby reducing the virulence of this pathogenic yeast. However, the current research is limited to computational methods and lacks experimental validation. In vitro and in vivo studies are needed to confirm these findings. Future investigations should include biochemical and cytotoxicity assays, animal models, and further characterization of the signaling pathways affected by KCG. To sum up, this research paves the way for a novel approach in targeting *C. albicans* QS and may further contribute to the development of antifungal therapies.

## Supplementary Information

Below is the link to the electronic supplementary material.

Supplementary Material 1

Supplementary Material 2

## Data Availability

All data generated or analyzed during this study are included in this published article.
