# Supplementary material for: Exploring propolis-derived compounds as quorum sensing inhibitors for Candida albicans: a molecular docking and dynamics simulations study
Source: Sci Rep. 2025 Sep 25;15:32899. doi: 10.1038/s41598-025-18001-1 (PMC12464178; doi:10.1038/s41598-025-18001-1)
Supplement: Supplementary file 1 — Supplementary Material 1 [file 41598_2025_18001_MOESM1_ESM.pdf]

Exploring Propolis-Derived Compounds as Quorum Sensing Inhibitors for *Candida albicans*: A Molecular Docking and MD Simulation Study

Fettouma Chraa[1], Doha EL Meskini[1], Ilham Kandoussi[1], Abdelhakim Bouyahya[2], Long Chiau Ming[3,4], Jactty Chew[3], Saad Moshawih[5], Rachid El Jaoudi[1], Mouna Ouadghiri[1], Tarik Aanniz[1*]

[1] Medical Biotechnology Laboratory (MedBiotech), Bioinova Research Center, Medical and Pharmacy School, Mohammed V University, Rabat, Morocco. (fettoumachraa04@gmail.com ; elmeskinidoha2@gmail.com ; i.kandoussi@um5r.ac.ma ; r.eljaoudi@um5r.ac.ma; m.ouadghiri@um5r.ac.ma ; t.aanniz@um5r.ac.ma)

[2] Laboratory of Human Pathologies Biology, Faculty of Sciences, Mohammed V[th] University in Rabat, Rabat, Morocco,

[3] Faculty of Medical and Life Sciences, Sunway University, Sunway City 47500, Malaysia

[4] Datta Meghe College of Pharmacy, Datta Meghe Institute of Higher Education and Research (deemed to be University), Sawangi (M), Wardha, India

[5] Faculty of Pharmacy, Al-Ahliyya Amman University, Amman, Jordan

*Corresponding Author: Tarik Aanniz, Medical Biotechnology Laboratory (MedBiotech), Bioinova Research Center, Medical and Pharmacy School, Mohammed Vth University, Rabat, 10102, Morocco, Email: t.aanniz@um5r.ac.ma

PROCHECK

Ramachandran Plot

saves

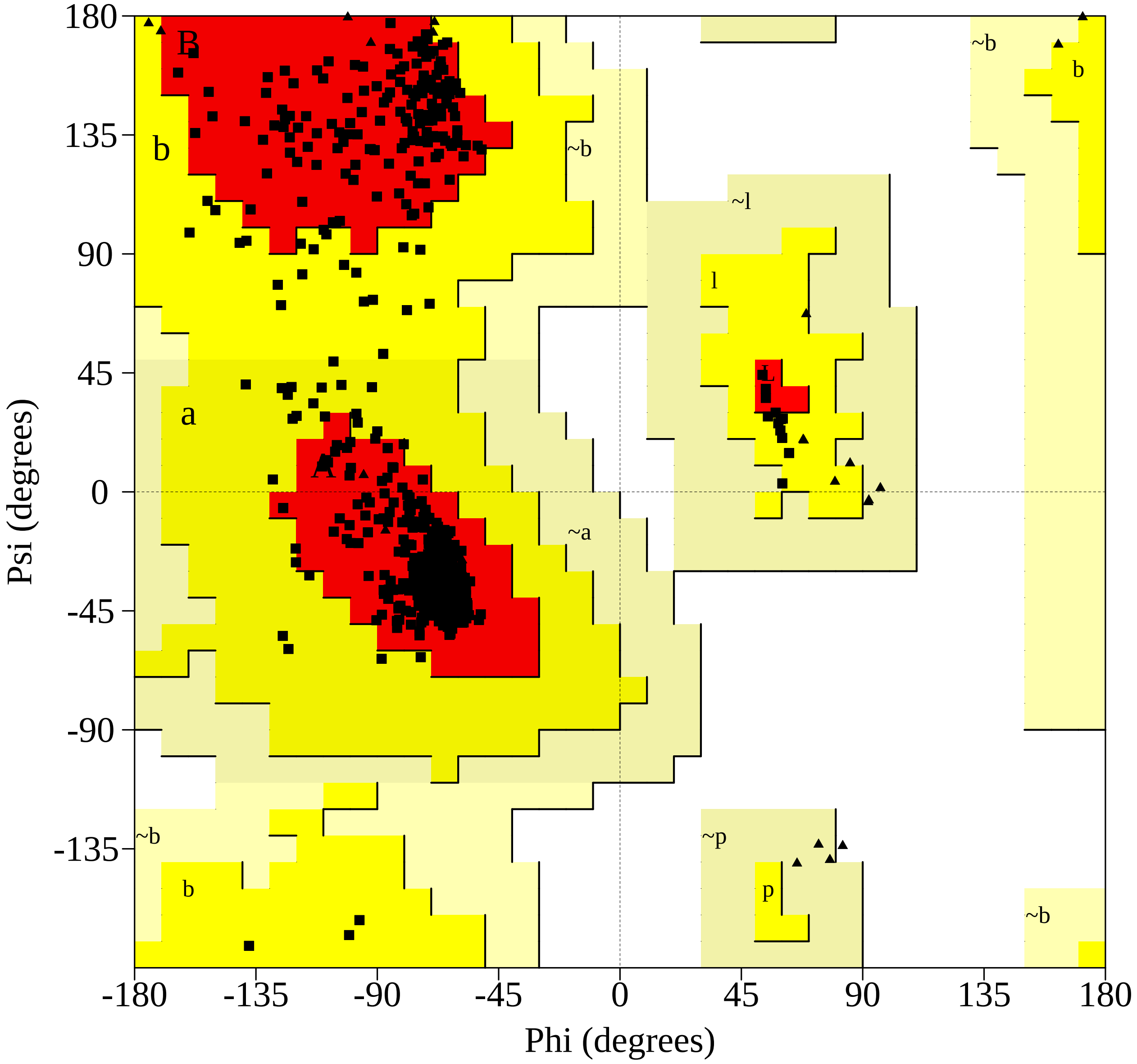

Plot statistics

Residues in most favoured regions [A,B,L]	704	92.6%
Residues in additional allowed regions [a,b,l,p]	56	7.4%
Residues in generously allowed regions [~a,~b,~l,~p]	0	0.0%
Residues in disallowed regions	0	0.0%
	----	------
Number of non-glycine and non-proline residues	760	100.0%
Number of end-residues (excl. Gly and Pro)	8	
Number of glycine residues (shown as triangles)	34	
Number of proline residues	38	

Total number of residues	840	

Based on an analysis of 118 structures of resolution of at least 2.0 Angstroms and R-factor no greater than 20%, a good quality model would be expected to have over 90% in the most favoured regions.

Ramachandran plots for all residue types

saves

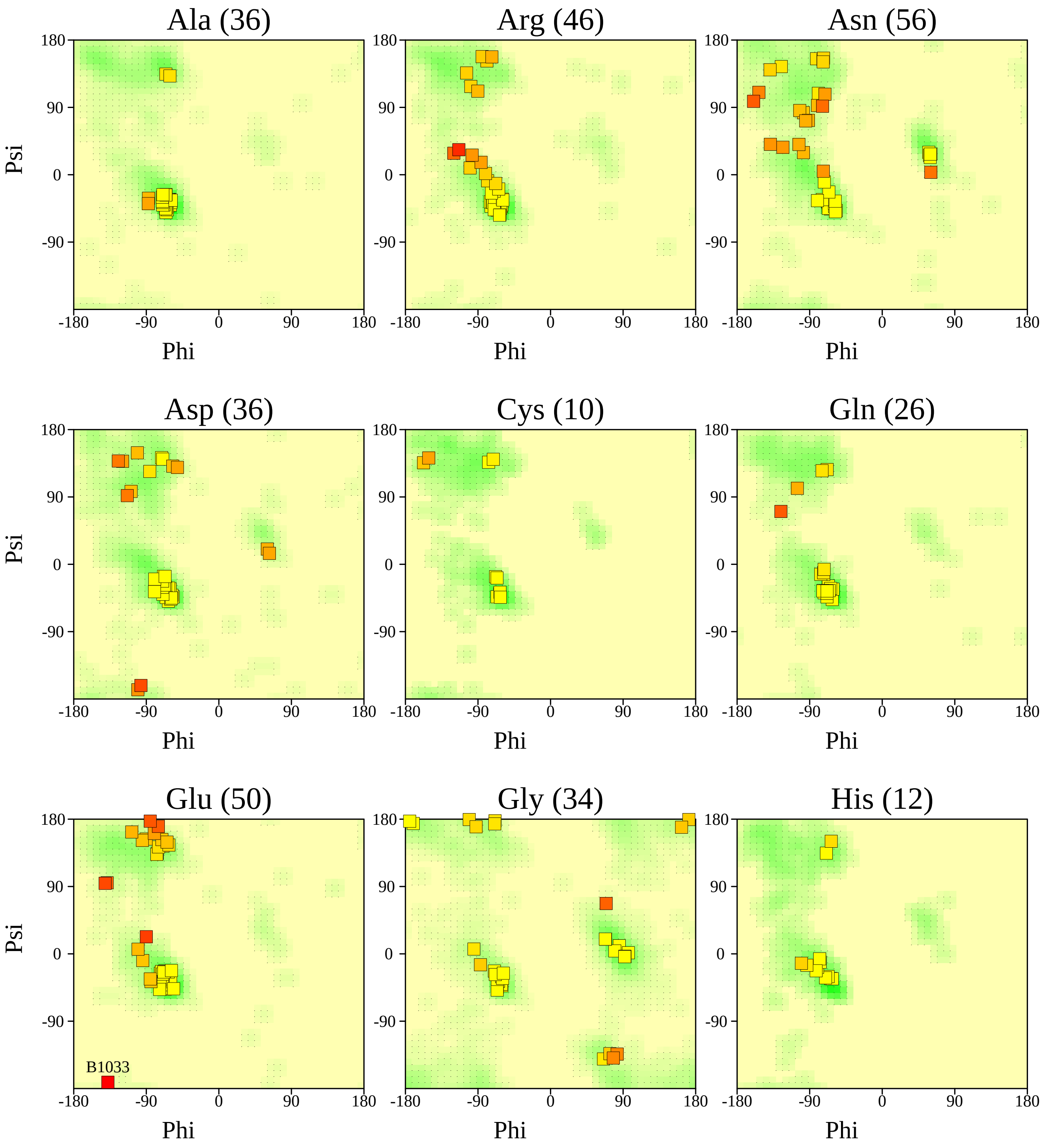

Numbers of residues are shown in brackets. Those in unfavourable conformations (score < -3.00) are labelled.
Shading shows favourable conformations as obtained from an analysis of 163 structures at resolution 2.0A or better.

Ramachandran plots for all residue types

saves

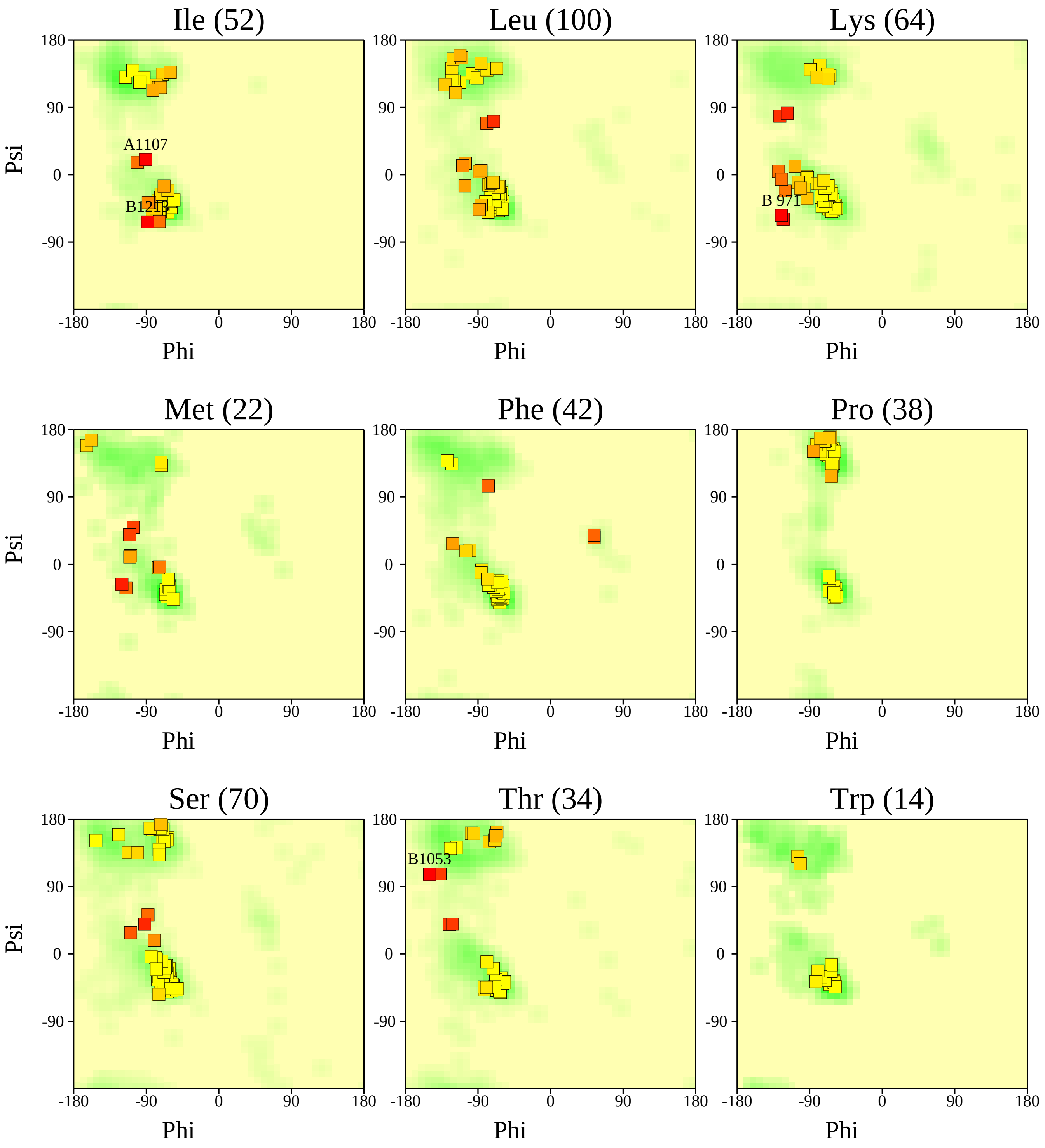

Numbers of residues are shown in brackets. Those in unfavourable conformations (score < -3.00) are labelled.
Shading shows favourable conformations as obtained from an analysis of 163 structures at resolution 2.0A or better.

Ramachandran plots for all residue types

saves

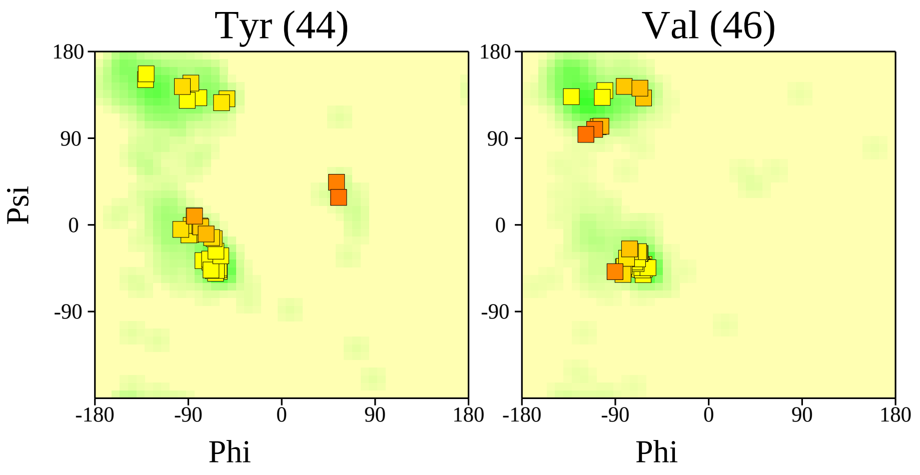

Numbers of residues are shown in brackets. Those in unfavourable conformations (score < -3.00) are labelled.
Shading shows favourable conformations as obtained from an analysis of 163 structures at resolution 2.0A or better.

PROCHECK

Chi1-Chi2 plots

saves

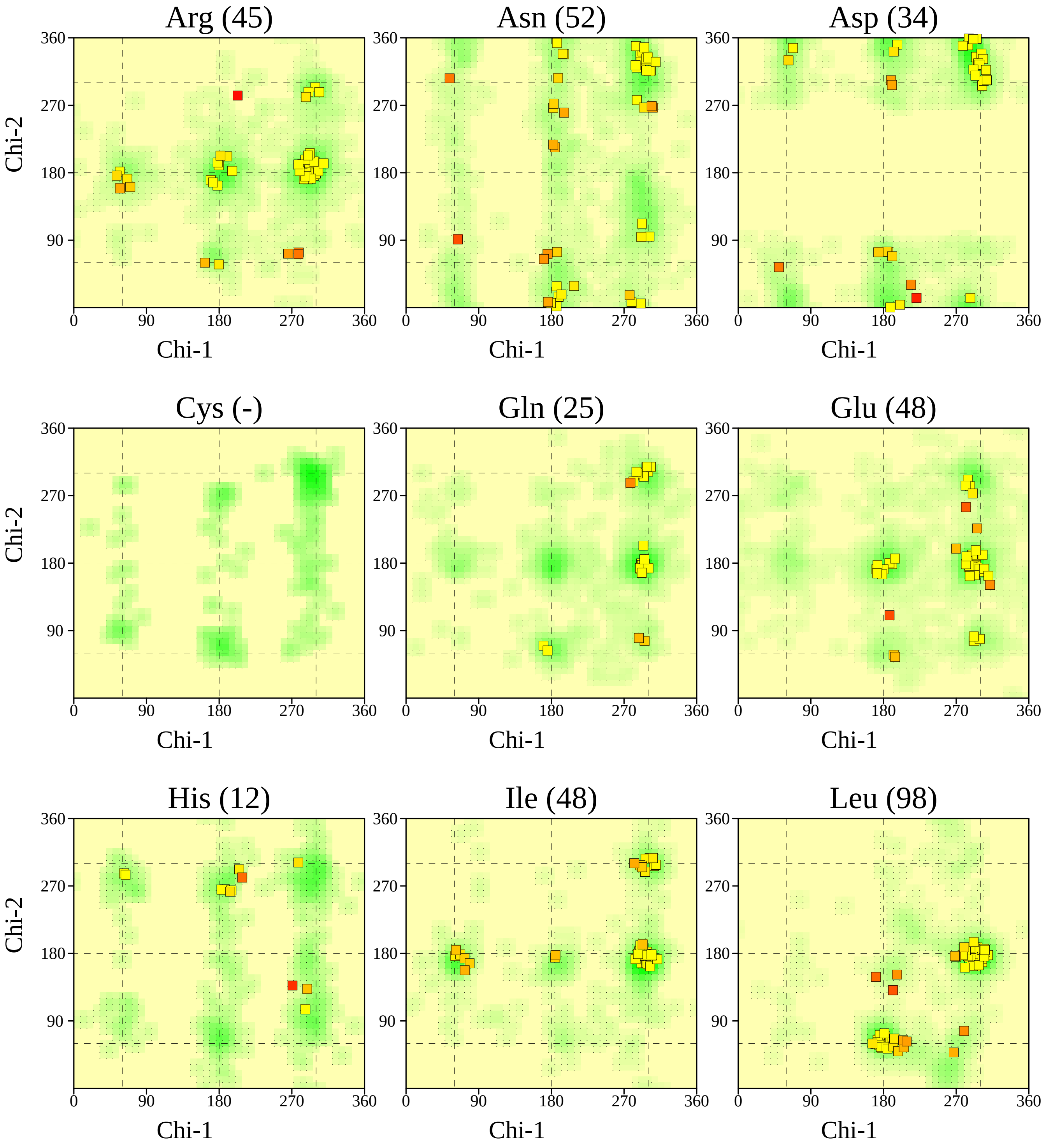

Numbers of residues are shown in brackets. Those in unfavourable conformations (score < -3.00) are labelled.
Shading shows favourable conformations as obtained from an analysis of 163 structures at resolution 2.0A or better.

Chi1-Chi2 plots

saves

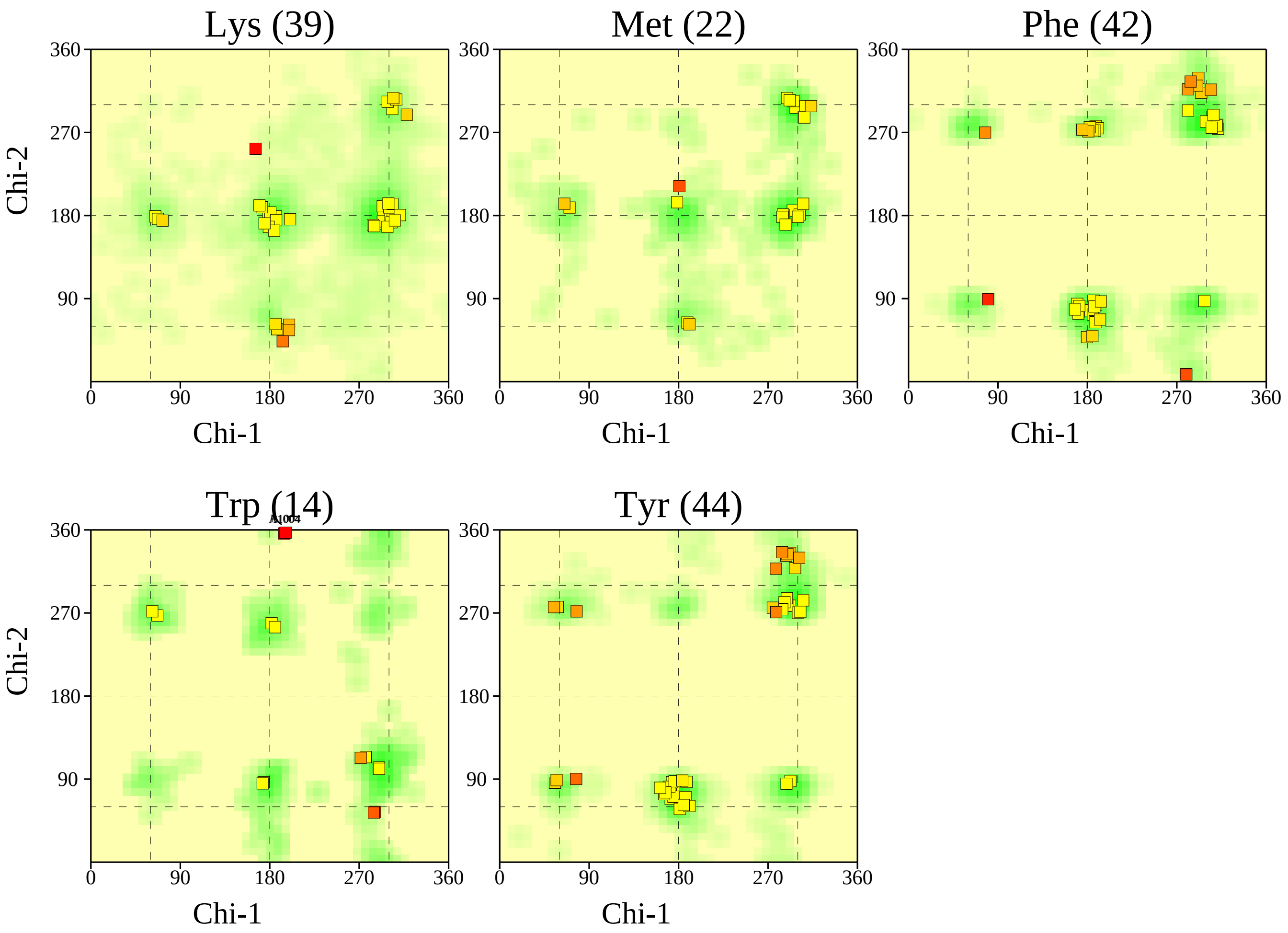

Numbers of residues are shown in brackets. Those in unfavourable conformations (score < -3.00) are labelled.
Shading shows favourable conformations as obtained from an analysis of 163 structures at resolution 2.0A or better.

PROCHECK

Main-chain parameters

saves

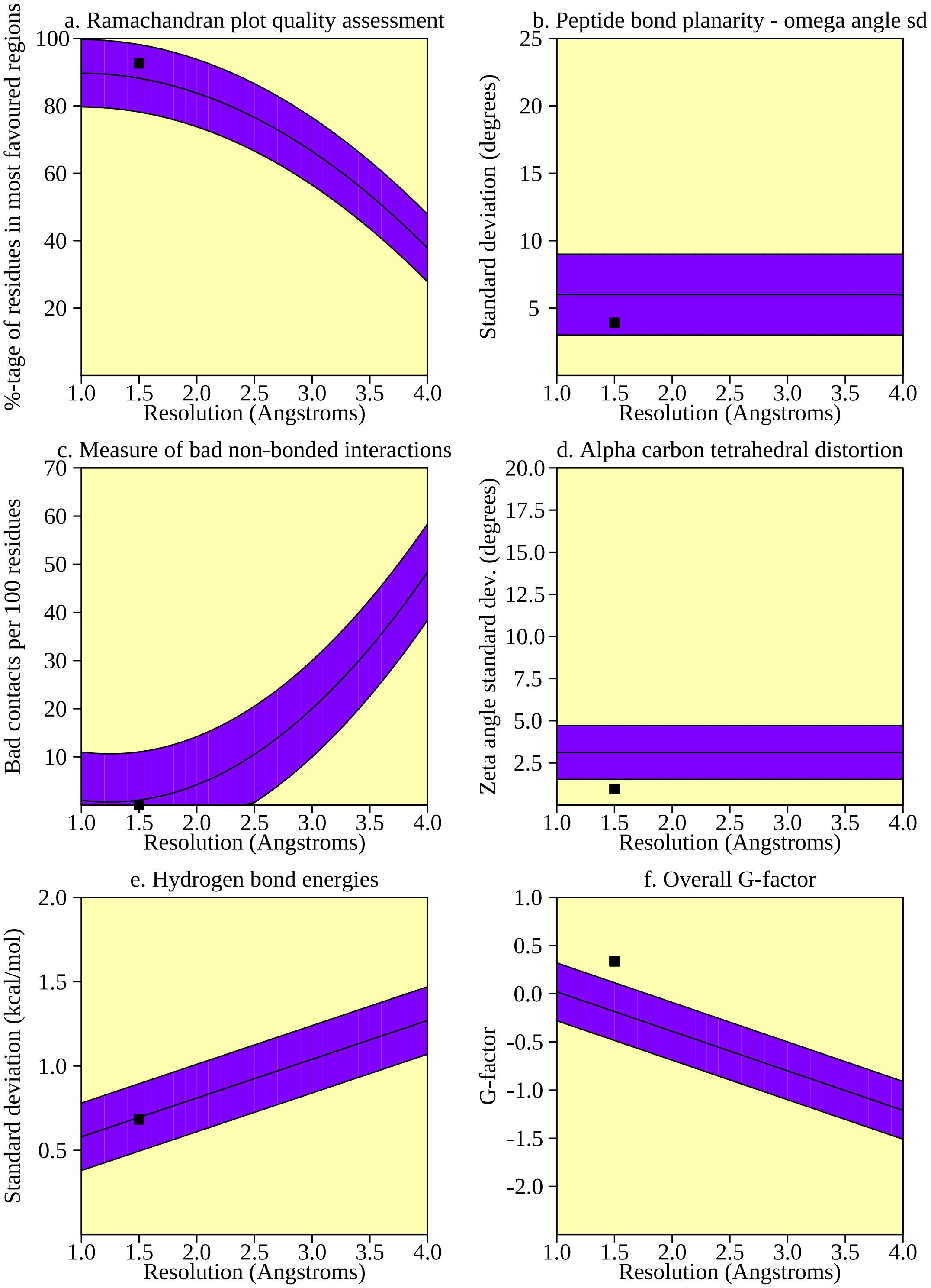

Plot statistics

Stereochemical parameter	No. of data pts	Parameter value	Comparison values Typical value	Band width	No. of band widths from mean	
a. %-tage residues in A, B, L	760	92.6	88.2	10.0	0.4	Inside
b. Omega angle st dev	836	3.9	6.0	3.0	-0.7	Inside
c. Bad contacts / 100 residues	0	0.0	1.0	10.0	-0.1	Inside
d. Zeta angle st dev	806	0.9	3.1	1.6	-1.4	BETTER
e. H-bond energy st dev	570	0.7	0.7	0.2	-0.1	Inside
f. Overall G-factor	840	0.3	-0.2	0.3	1.7	BETTER

Residue properties

saves

a. Absolute deviation from mean Chi-1 value (excl. Pro)

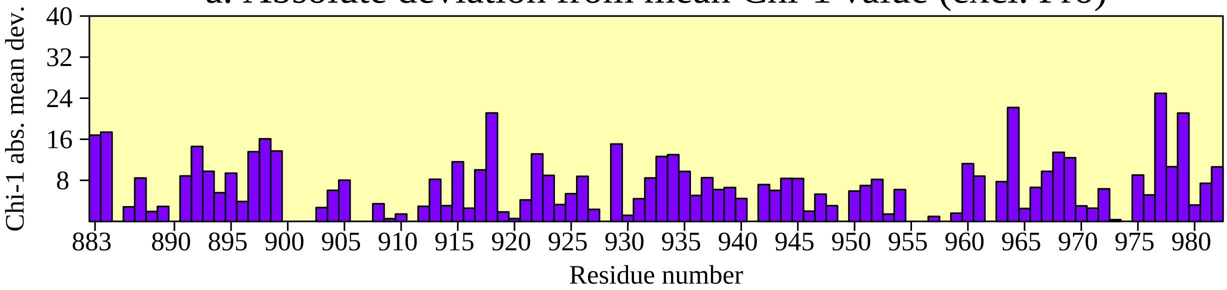

b. Absolute deviation from mean of omega torsion

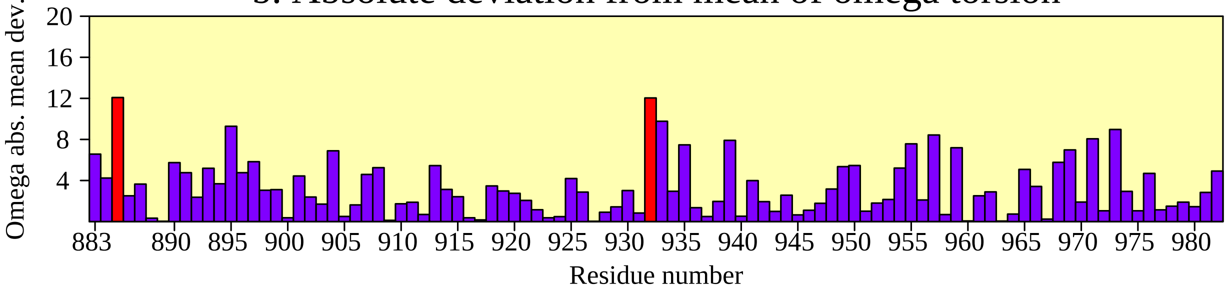

c. C-alpha chirality: abs. deviation of zeta torsion

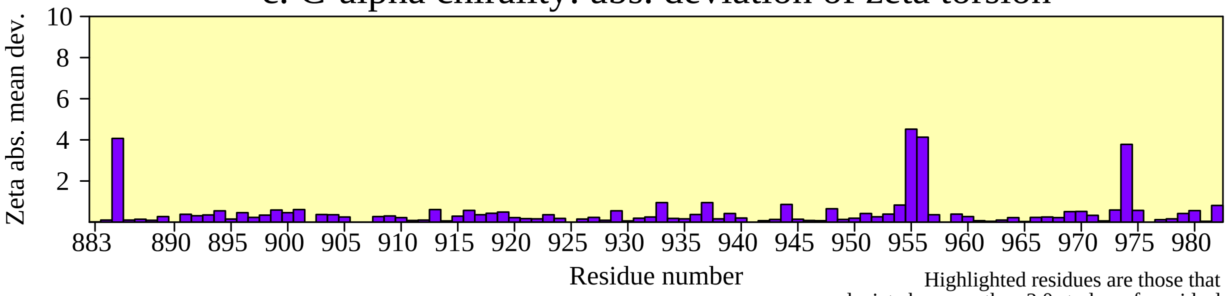

Highlighted residues are those that deviate by more than 2.0 st. devs. from ideal

d. Secondary structure & estimated accessibility

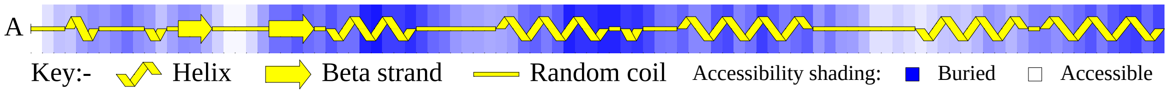

e. Sequence & Ramachandran regions

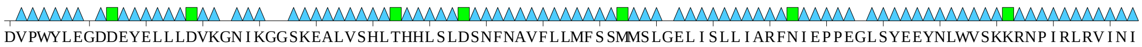

f. Max. deviation (see listing)

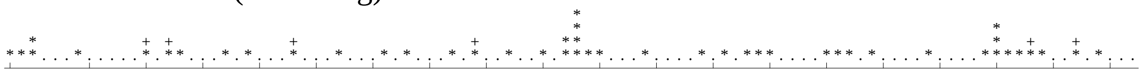

g. G-factors

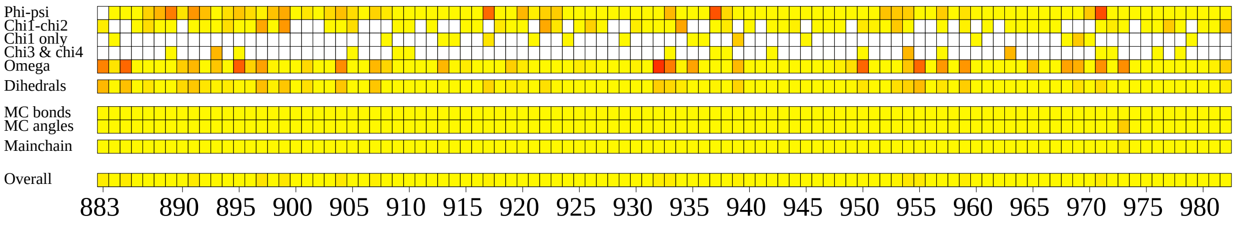

Residue properties

saves

a. Absolute deviation from mean Chi-1 value (excl. Pro)

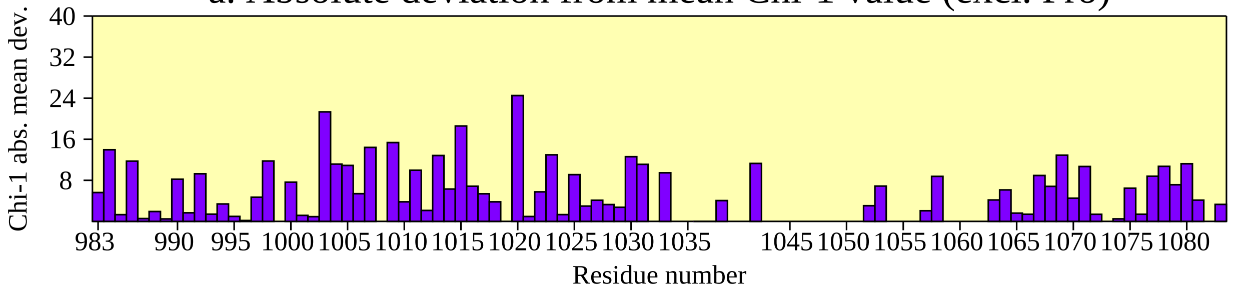

b. Absolute deviation from mean of omega torsion

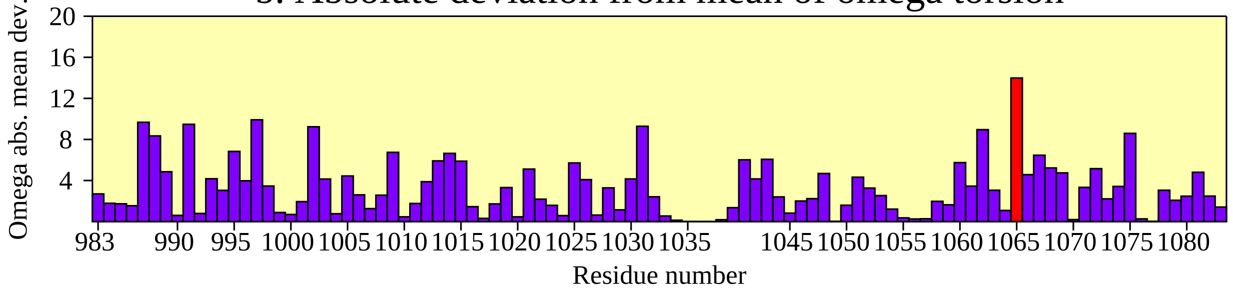

c. C-alpha chirality: abs. deviation of zeta torsion

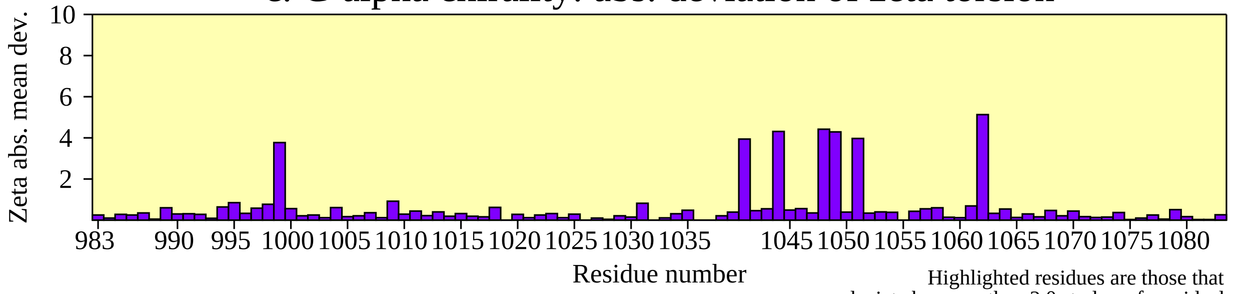

Highlighted residues are those that deviate by more than 2.0 st. devs. from ideal

d. Secondary structure & estimated accessibility

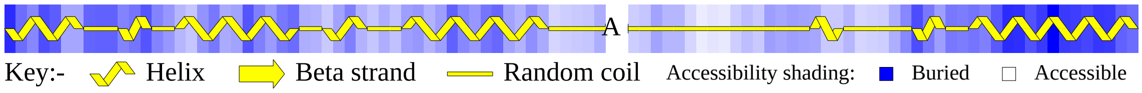

e. Sequence & Ramachandran regions

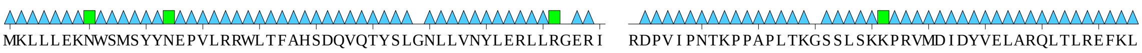

f. Max. deviation (see listing)

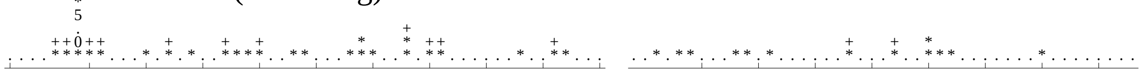

g. G-factors

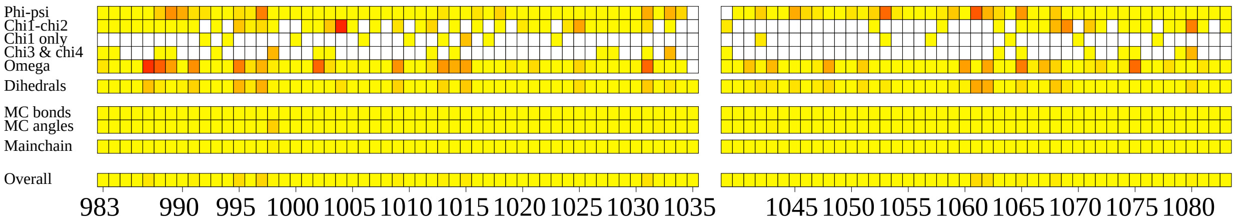

Residue properties

saves

a. Absolute deviation from mean Chi-1 value (excl. Pro)

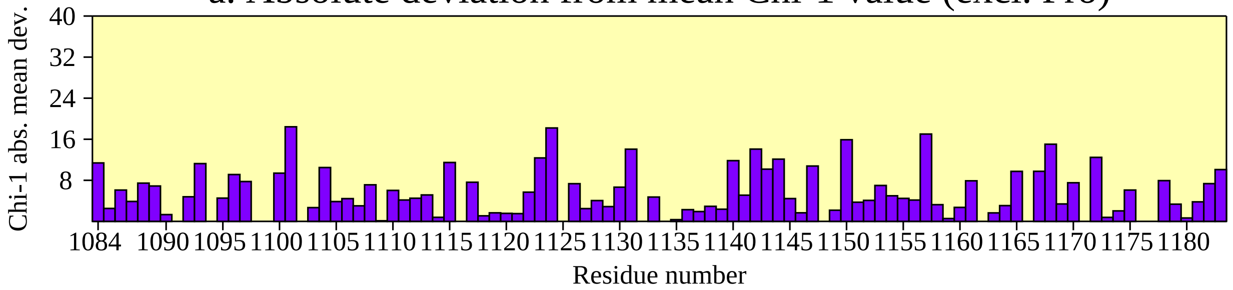

b. Absolute deviation from mean of omega torsion

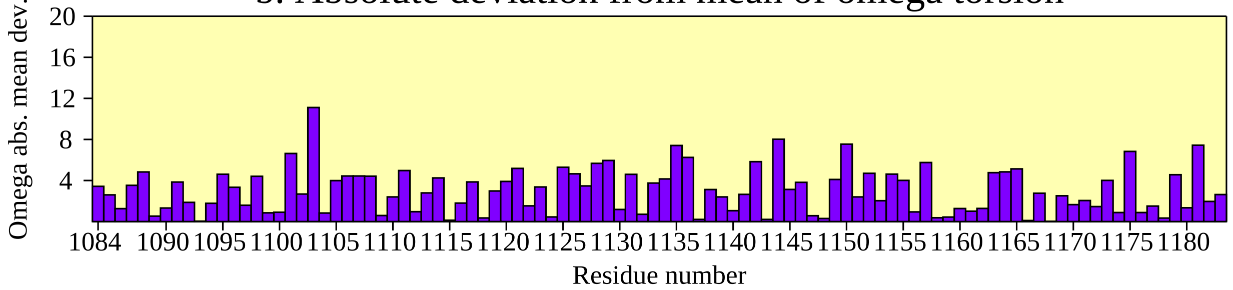

c. C-alpha chirality: abs. deviation of zeta torsion

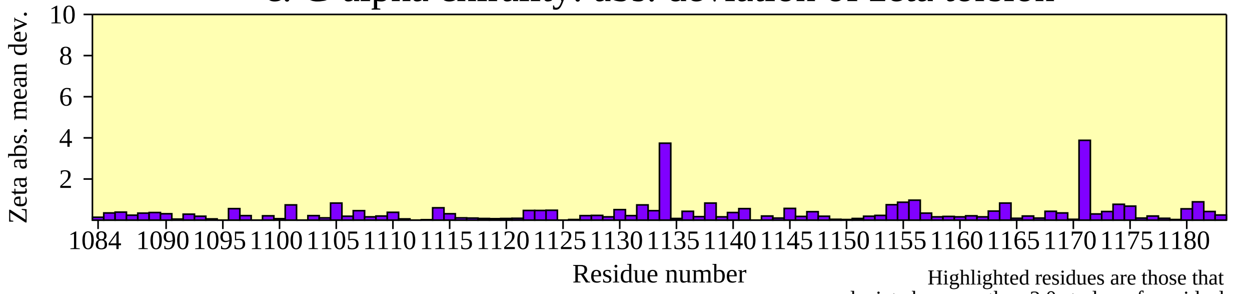

Highlighted residues are those that deviate by more than 2.0 st. devs. from ideal

d. Secondary structure & estimated accessibility

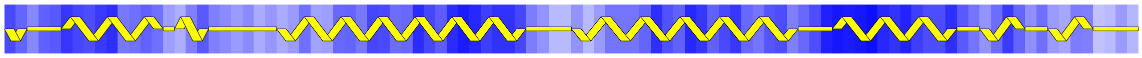

Key:- Helix Beta strand Random coil Accessibility shading: Buried Accessible

e. Sequence & Ramachandran regions

Most favoured Allowed Generous Disallowed

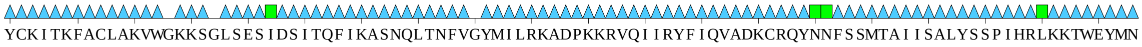

f. Max. deviation (see listing)

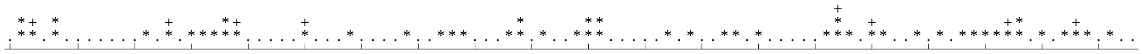

g. G-factors

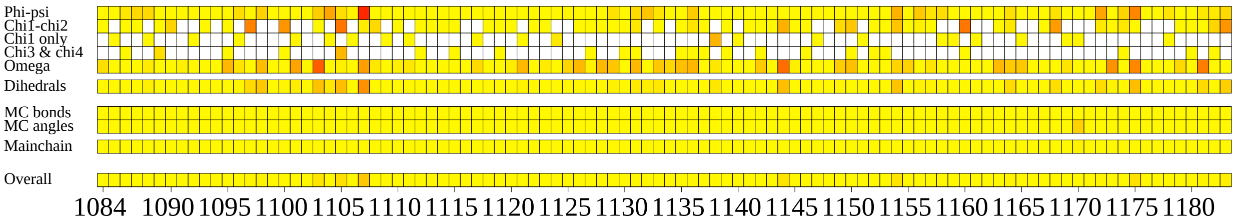

Residue properties

saves

a. Absolute deviation from mean Chi-1 value (excl. Pro)

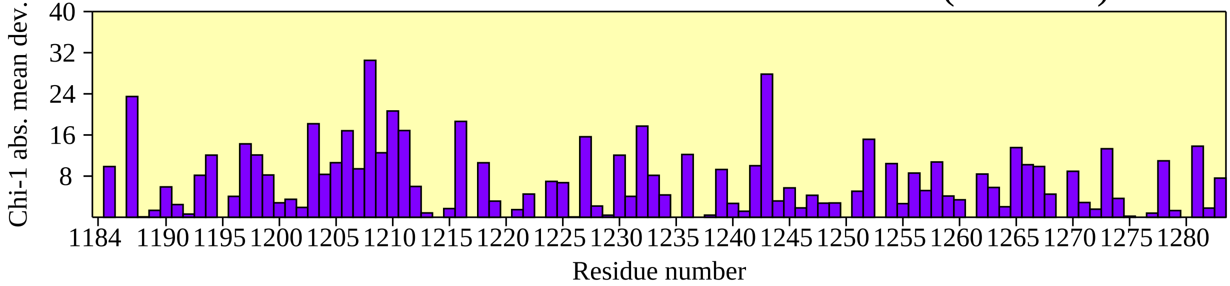

b. Absolute deviation from mean of omega torsion

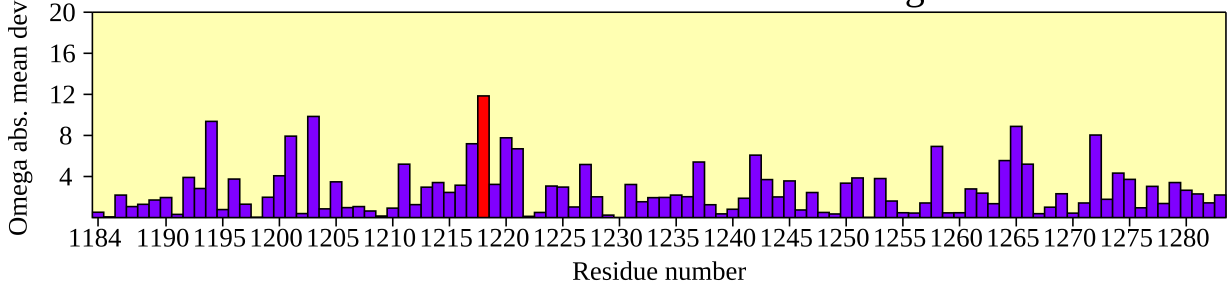

c. C-alpha chirality: abs. deviation of zeta torsion

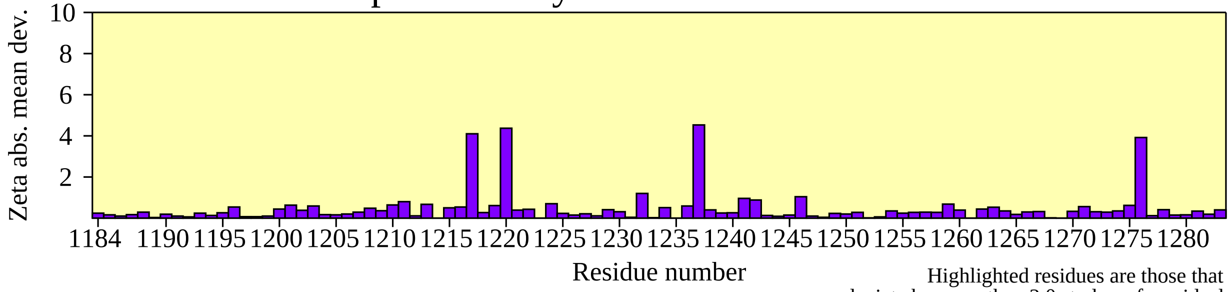

Highlighted residues are those that deviate by more than 2.0 st. devs. from ideal

d. Secondary structure & estimated accessibility

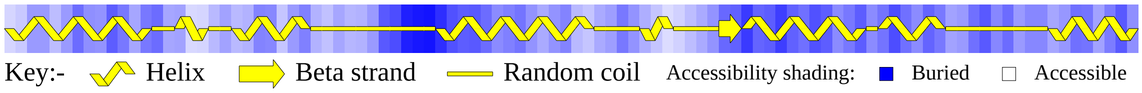

e. Sequence & Ramachandran regions

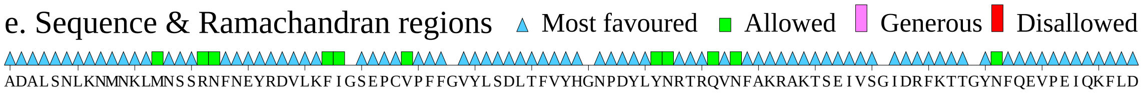

f. Max. deviation (see listing)

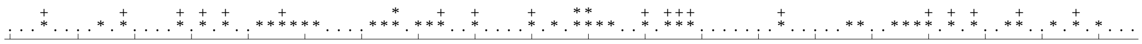

g. G-factors

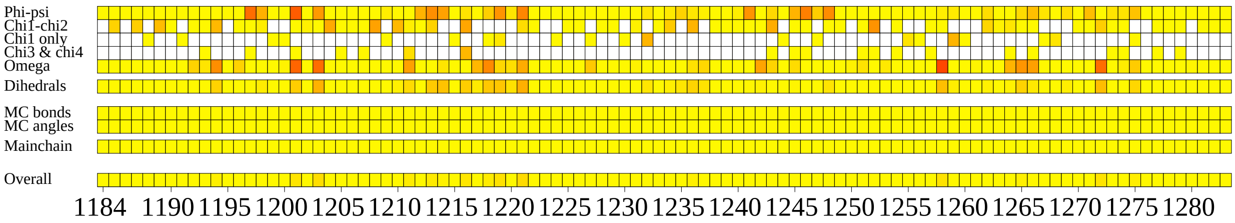

Residue properties

saves

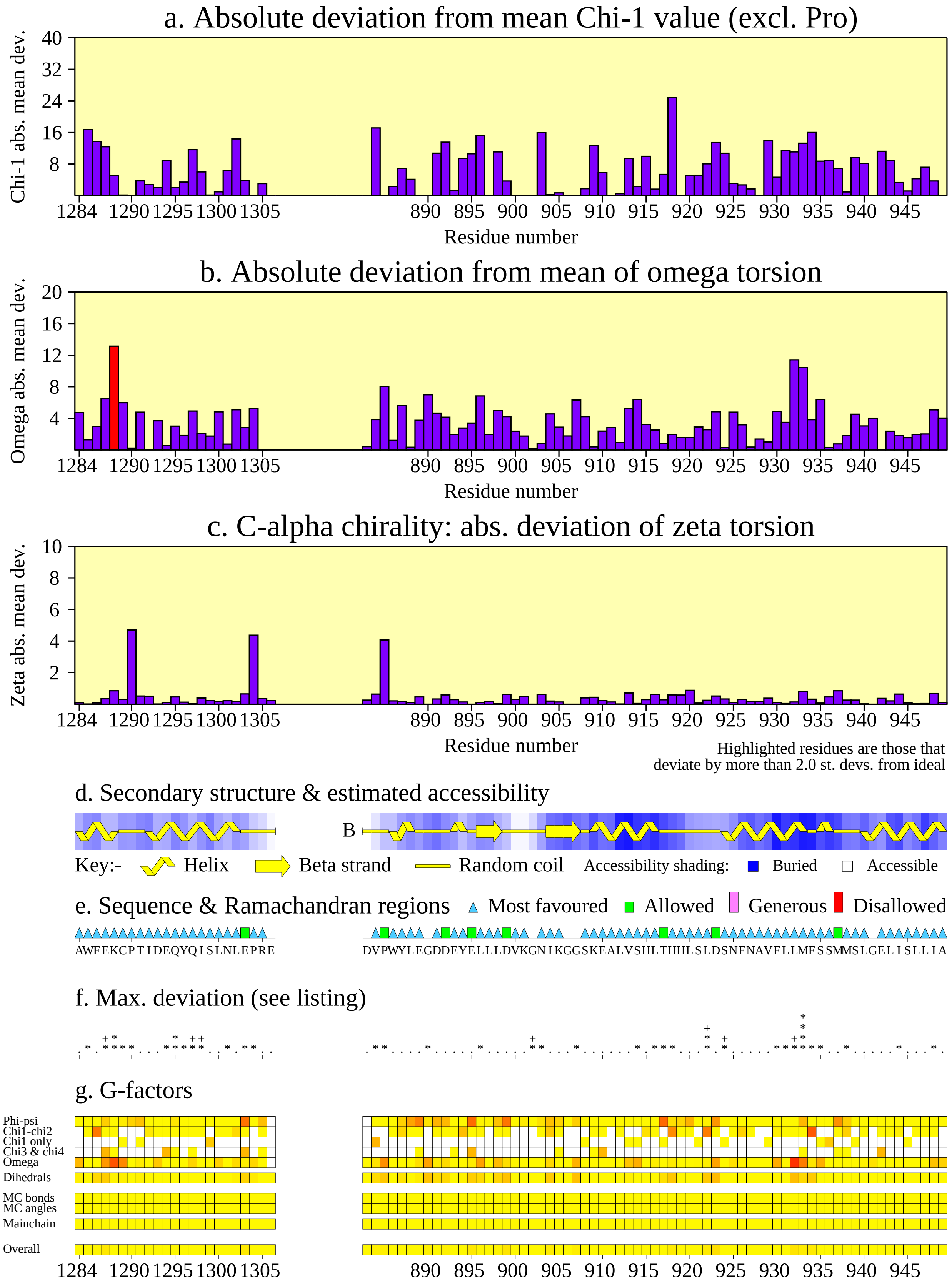

PROCHECK

Residue properties

saves

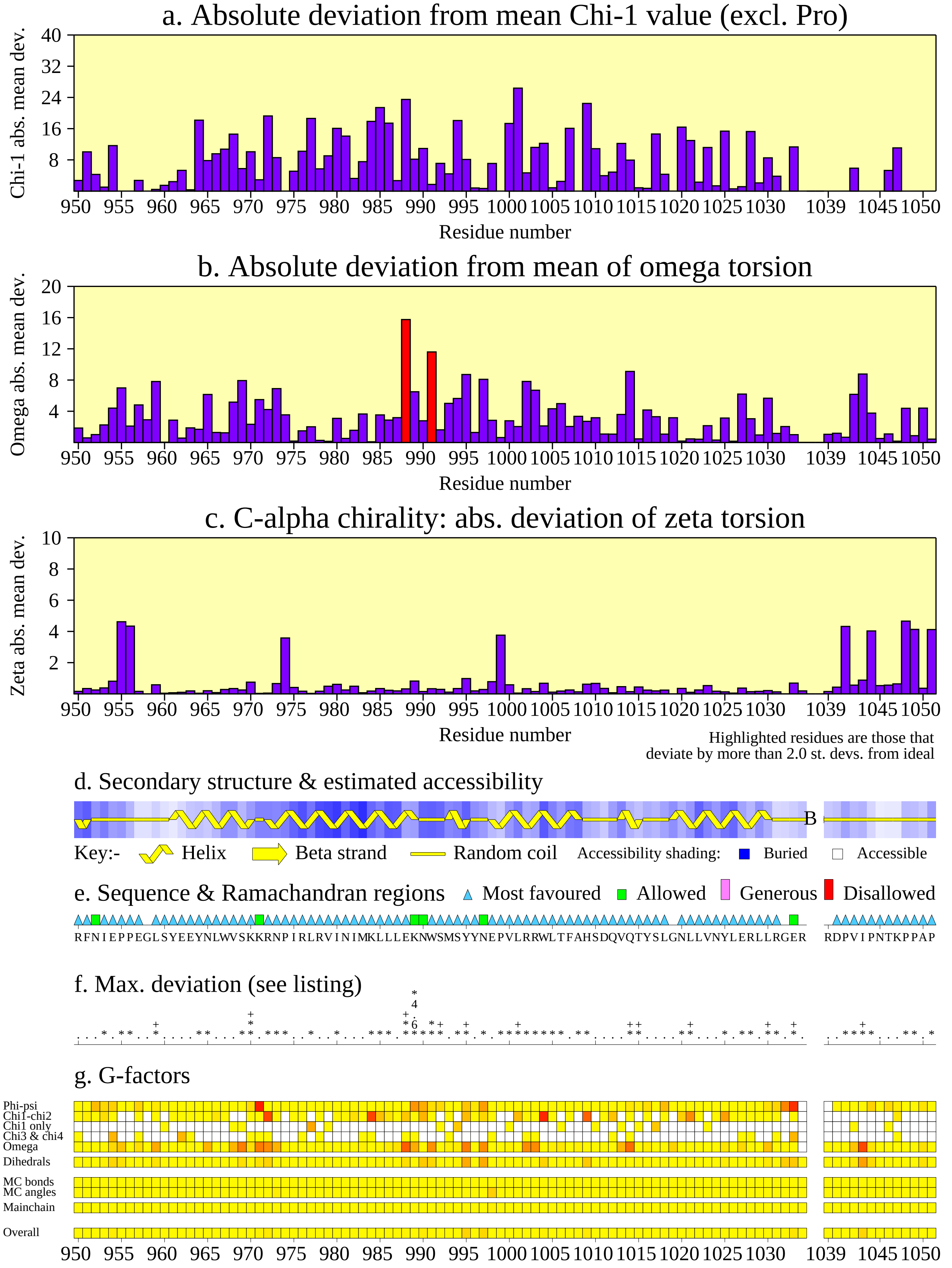

Residue properties

saves

a. Absolute deviation from mean Chi-1 value (excl. Pro)

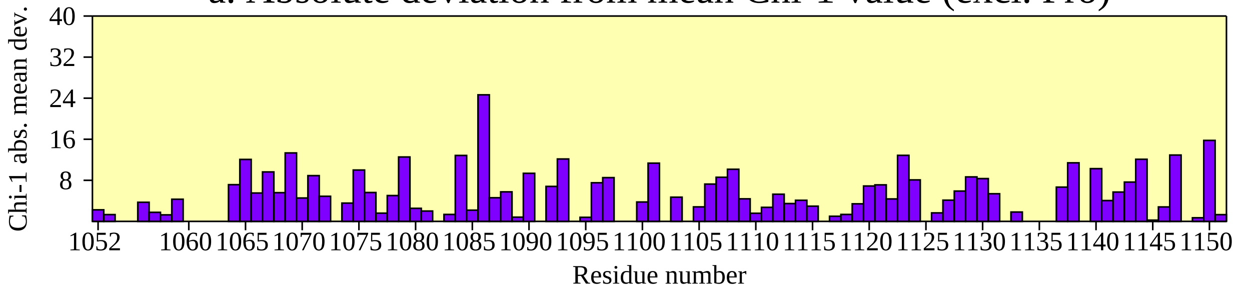

b. Absolute deviation from mean of omega torsion

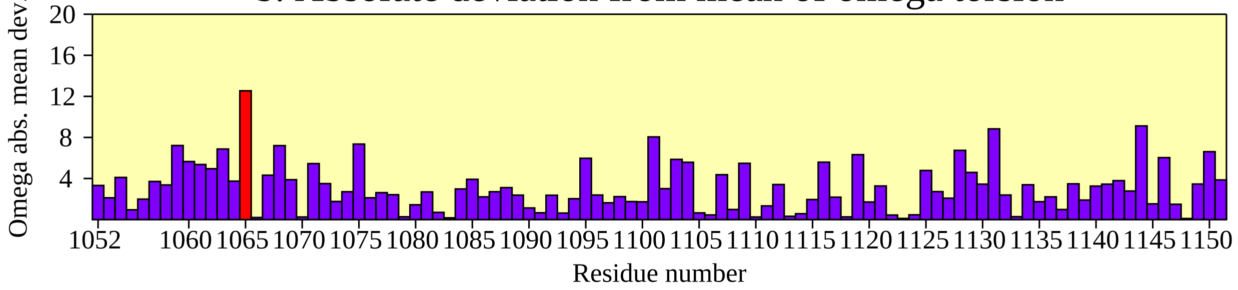

c. C-alpha chirality: abs. deviation of zeta torsion

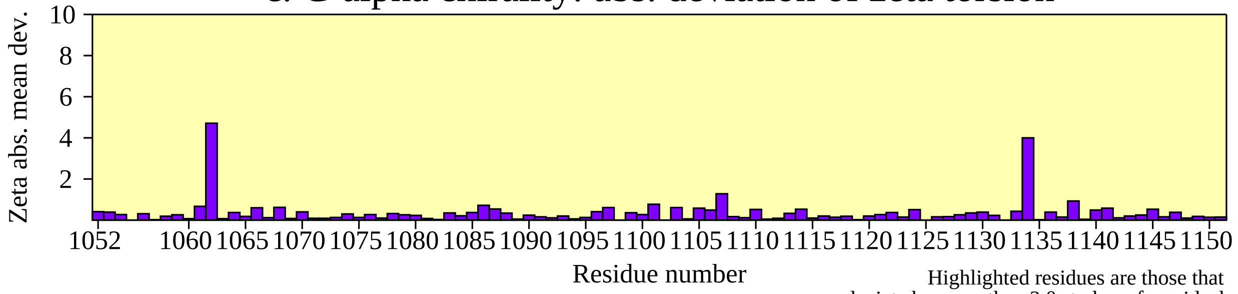

Highlighted residues are those that deviate by more than 2.0 st. devs. from ideal

d. Secondary structure & estimated accessibility

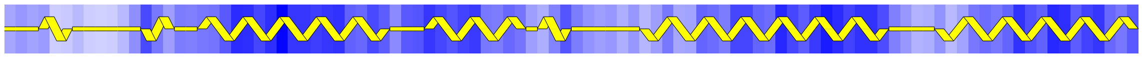

Key:- Helix Beta strand Random coil Accessibility shading: Buried Accessible

e. Sequence & Ramachandran regions

Most favoured Allowed Generous Disallowed

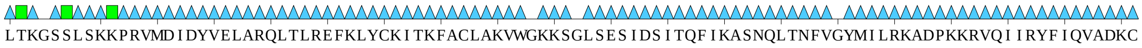

f. Max. deviation (see listing)

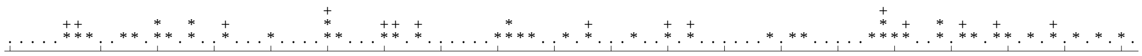

g. G-factors

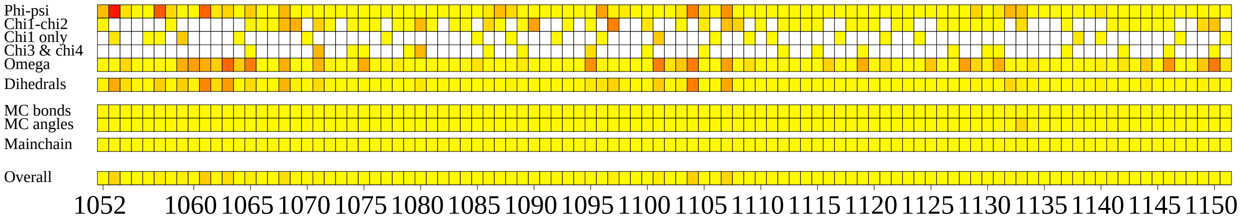

PROCHECK

Residue properties

saves

a. Absolute deviation from mean Chi-1 value (excl. Pro)

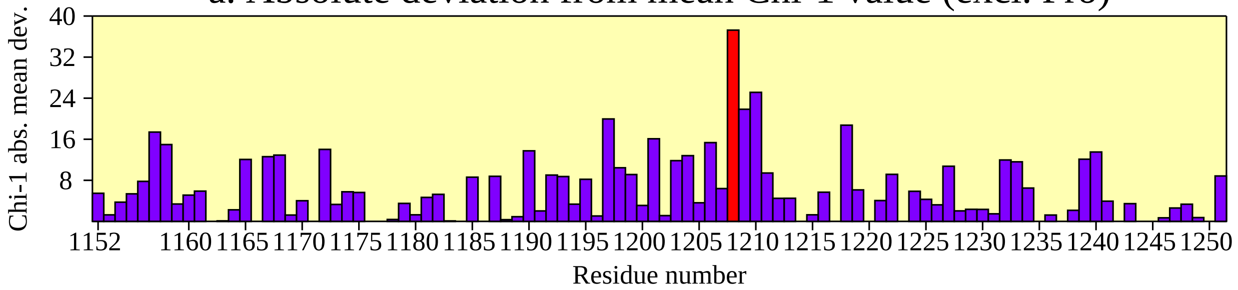

b. Absolute deviation from mean of omega torsion

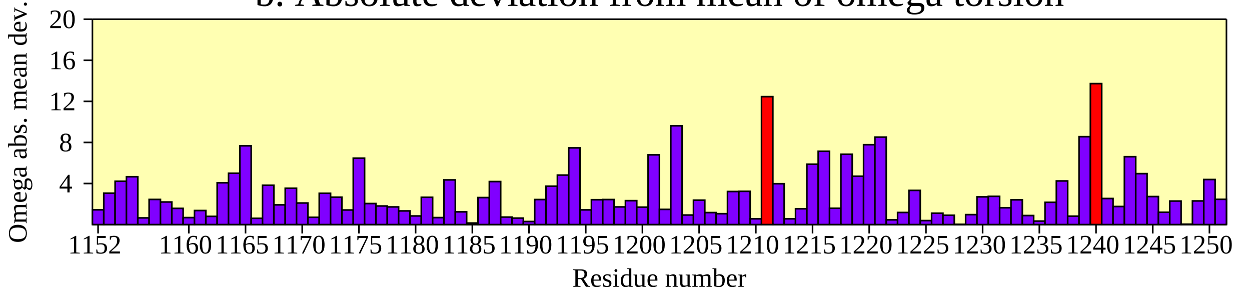

c. C-alpha chirality: abs. deviation of zeta torsion

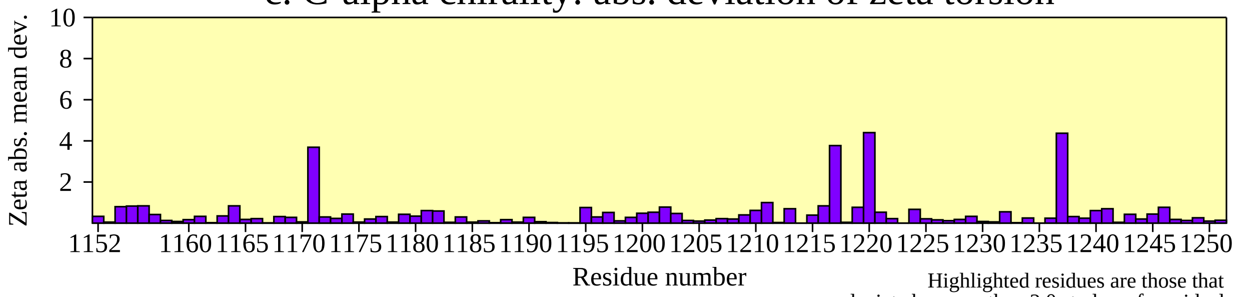

Highlighted residues are those that deviate by more than 2.0 st. devs. from ideal

d. Secondary structure & estimated accessibility

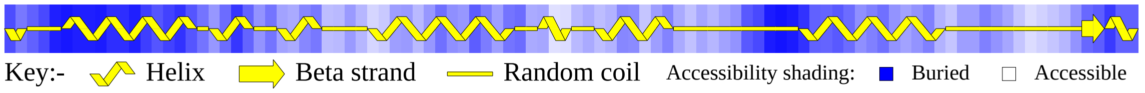

e. Sequence & Ramachandran regions

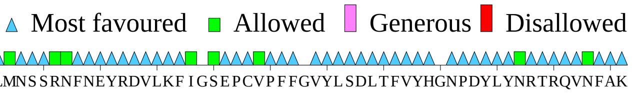

f. Max. deviation (see listing)

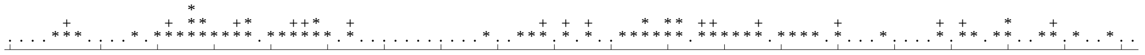

g. G-factors

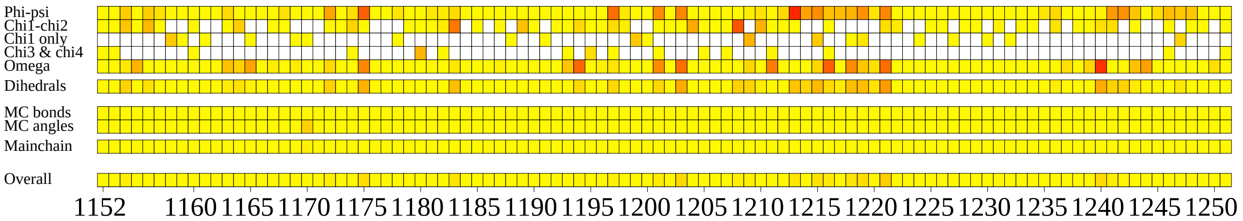

Residue properties

saves

a. Absolute deviation from mean Chi-1 value (excl. Pro)

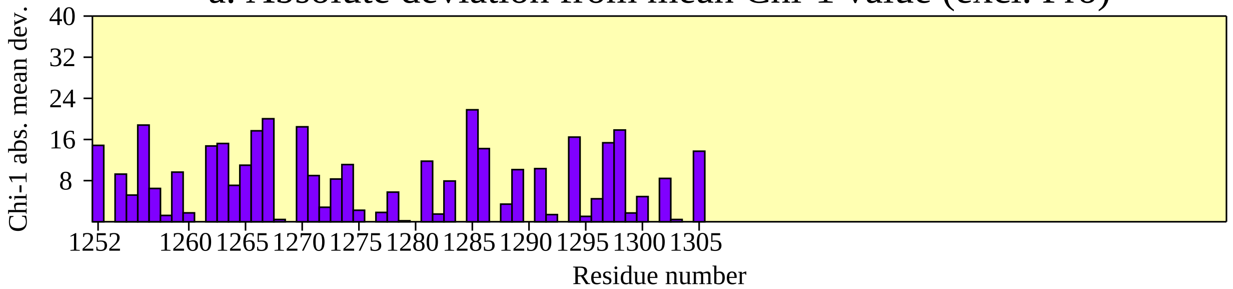

b. Absolute deviation from mean of omega torsion

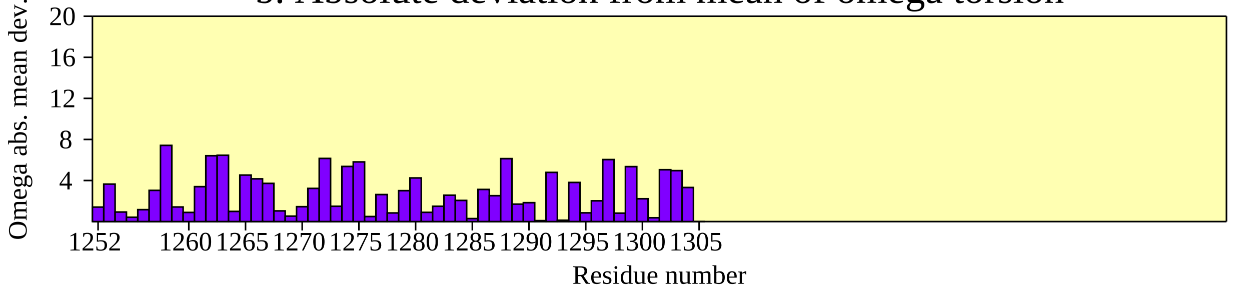

c. C-alpha chirality: abs. deviation of zeta torsion

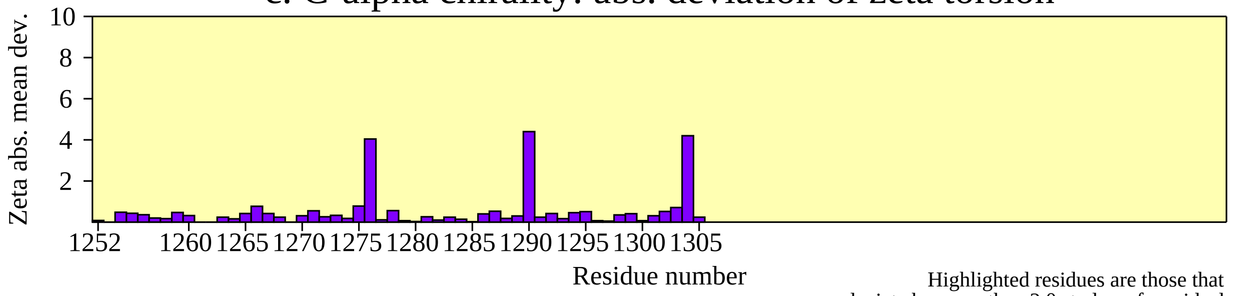

Highlighted residues are those that deviate by more than 2.0 st. devs. from ideal

d. Secondary structure & estimated accessibility

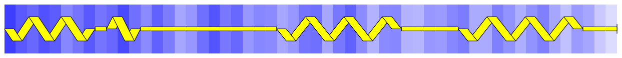

Key:- Helix Beta strand Random coil Accessibility shading: Buried Accessible

e. Sequence & Ramachandran regions

Most favoured Allowed Generous Disallowed

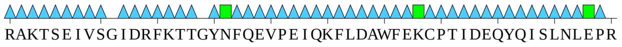

f. Max. deviation (see listing)

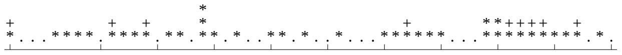

g. G-factors

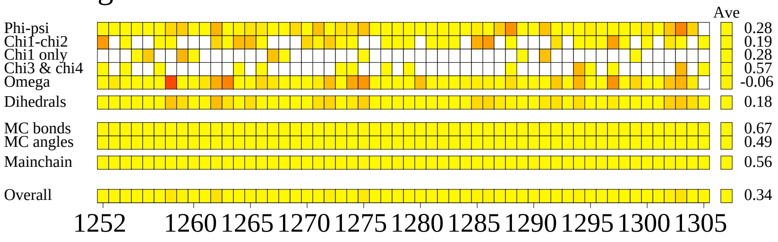

RMS distances from planarity

saves

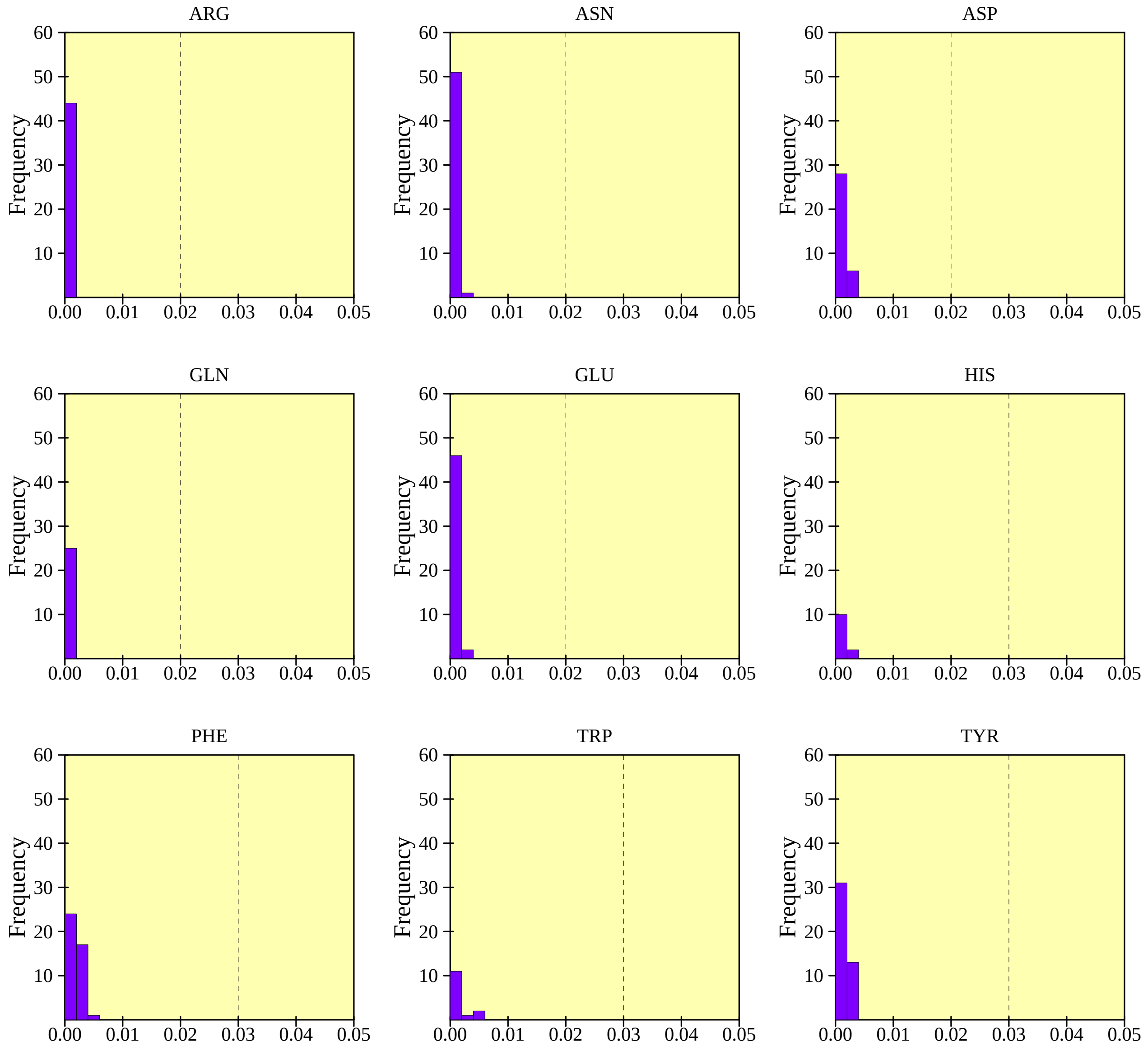

Histograms showing RMS distances of planar atoms from best-fit plane.
Black bars indicate large deviations from planarity: RMS dist > 0.03 for rings, and > 0.02 otherwise.
