# Supplementary material for: Exploring propolis-derived compounds as quorum sensing inhibitors for Candida albicans: a molecular docking and dynamics simulations study
Source: Sci Rep. 2025 Sep 25;15:32899. doi: 10.1038/s41598-025-18001-1 (PMC12464178; doi:10.1038/s41598-025-18001-1)
Supplement: Supplementary file 2 — Supplementary Material 2 [file 41598_2025_18001_MOESM2_ESM.docx]

Exploring Propolis-Derived Compounds as Quorum Sensing Inhibitors for *Candida albicans*: A Molecular Docking and MD Simulation Study

Fettouma Chraa^1^, Doha EL Meskini^1^, Ilham Kandoussi^1^, Abdelhakim Bouyahya^2^, Long Chiau Ming^3,4^, Jactty Chew^3^, Saad Moshawih^5^, Rachid El Jaoudi^1^, Mouna Ouadghiri^1^, Tarik Aanniz^1*^

1 Medical Biotechnology Laboratory (MedBiotech), Bioinova Research Center, Medical and Pharmacy School, Mohammed V University, Rabat, Morocco. [(fettoumachraa04@gmail.com](mailto:(fettoumachraa04@gmail.com)

; [elmeskinidoha2@gmail.com](mailto:elmeskinidoha2@gmail.com) ; [i.kandoussi@um5r.ac.ma](mailto:i.kandoussi@um5r.ac.ma) ; [r.eljaoudi@um5r.ac.ma;](mailto:r.eljaoudi@um5r.ac.ma) [m.ouadghiri@um5r.ac.ma](mailto:m.ouadghiri@um5r.ac.ma) ; [t.aanniz@um5r.ac.ma)](mailto:t.aanniz@um5r.ac.ma)

2 Laboratory of Human Pathologies Biology, Faculty of Sciences, Mohammed V^th^ University in Rabat, Rabat, Morocco,

3 Faculty of Medical and Life Sciences, Sunway University, Sunway City 47500, Malaysia

4 Datta Meghe College of Pharmacy, Datta Meghe Institute of Higher Education and Research (deemed to be University), Sawangi (M), Wardha, India

5 Faculty of Pharmacy, Al-Ahliyya Amman University, Amman, Jordan

*Corresponding Author: Tarik Aanniz, Medical Biotechnology Laboratory (MedBiotech), Bioinova Research Center, Medical and Pharmacy School, Mohammed Vth University, Rabat, 10102, Morocco, Email: [t.aanniz@um5r.ac.ma](mailto:t.aanniz@um5r.ac.ma)

**Supplementary Table 1.** Docking binding energies (kcal/mol) of selected Propolis-Derived compounds against CYC and RAS1 receptors along with their 2D structures in *C. albicans.*

**Compound Name Structure Binding Energy (CYC)**

**Binding Energy (RAS1)**

**Key interactions**


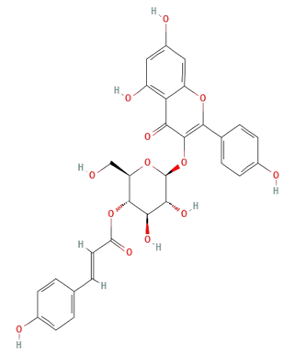

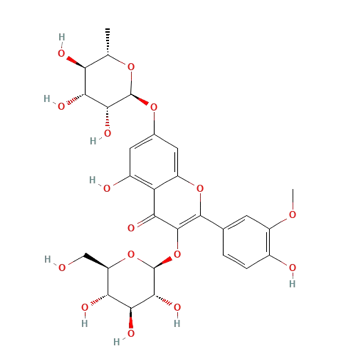

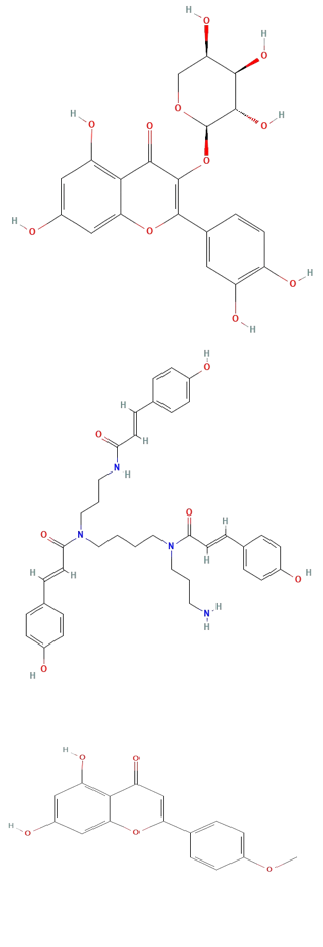


**KCG**

-10

-9.4

H-bonds, Pi-Cation, Pi- Alkyl, Pi-Pi T-shaped, Amide-Pi Stacked, van der Waals

**IGR**

-9.0

-8.4

H-bonds, Pi-Cation, Pi- Anion, Pi-Alkyl, Carbon hydrogen bond

**Quercetin-3-O- arabinoside**

-7.9

-7.8

Pi-anion, Pi-alkyl, Pi-Pi stacked

**N1,N5,N10-Tris-**

**trans-p- coumaroylspermin e**

-7.0

-7.8

Pi-Alkyl, Pi-anion, Pi- cation

**Acacetin**

-7.6

-7.5

Pi-cation, Pi-Pi stacked


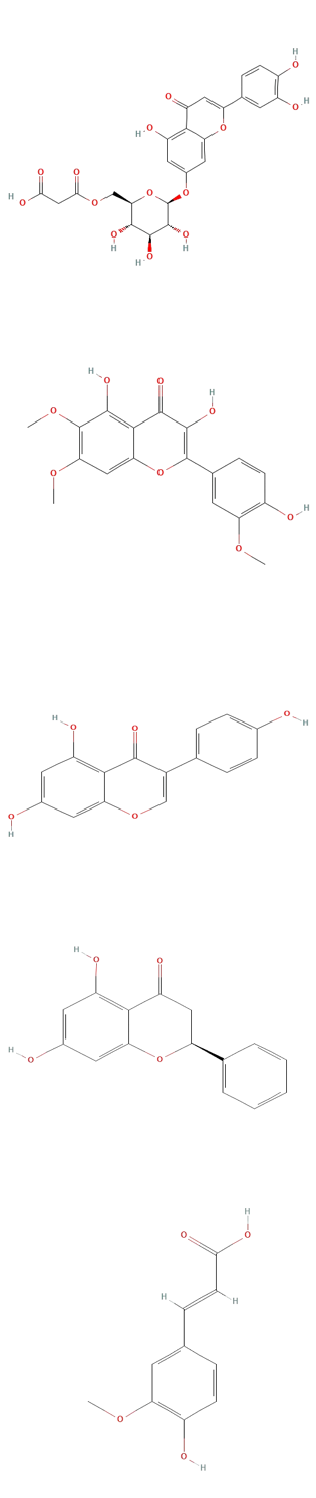


**Luteolin 7-O-(6 ''-**

**malonylglucoside)**

-7.0

-7.5

Pi-anion, Pi-alkyl, Pi-Pi stacked

**3,4’,5-Trihydroxy- 3’,6,7-**

**trimethoxyflavone**

-7.6

-7.3

Pi-Alkyl, Pi-anion, Pi- cation

**Genistein**

-7.4

-7.2

Pi-Pi T-shaped, Amide-Pi stacked, Pi-Alkyl

**Pinocembrin**

-7.1

-7.1

Pi-Alkyl, Pi-anion, Pi- cation

**Ferulic acid**

-7.1

-7.0

Pi-Pi T-shaped, Amide-Pi stacked, Pi-Alkyl


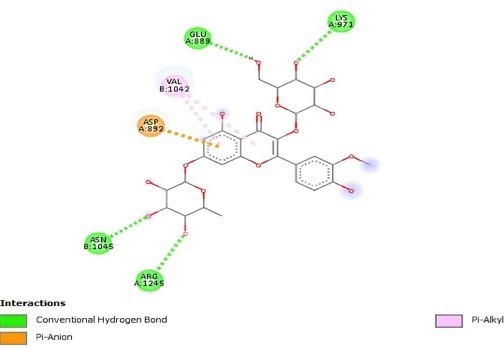
**Supplementary Table 2.** Visualization of binding interactions between Moroccan propolis compounds and *C. albicans* quorum sensing receptors in 2D and 3D representations.

**Protein-ligand 2D pose 3D pose CYC-KCG**

**
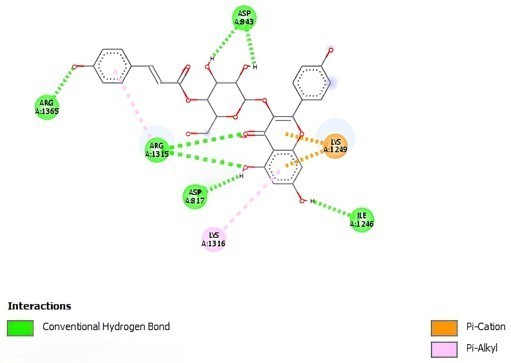

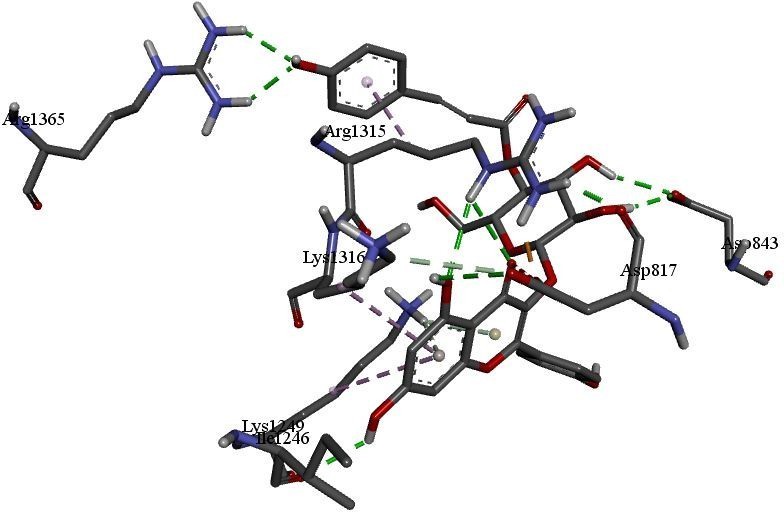
**

**RAS1-KCG**


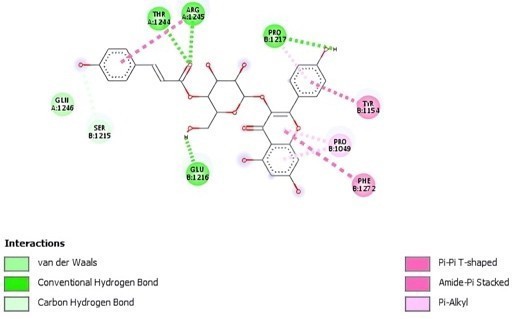

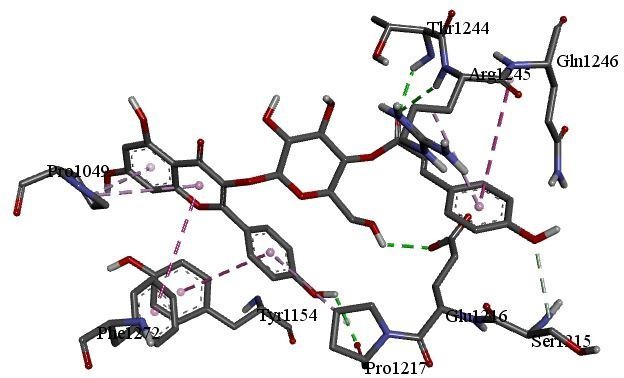


**CYC-IGR**


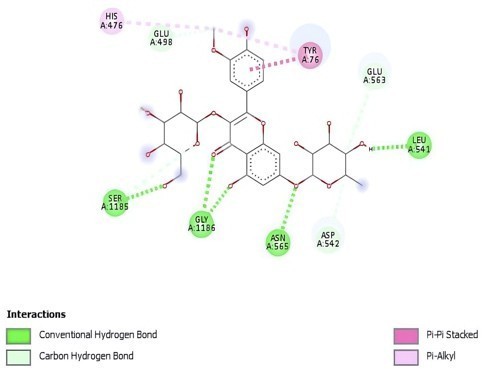

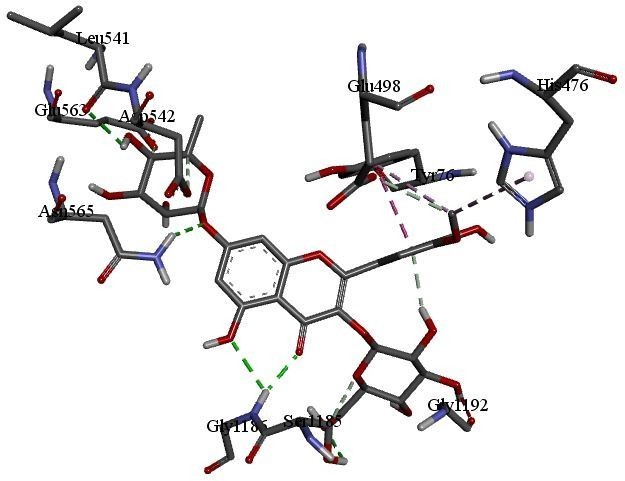


**RAS1-IGR**


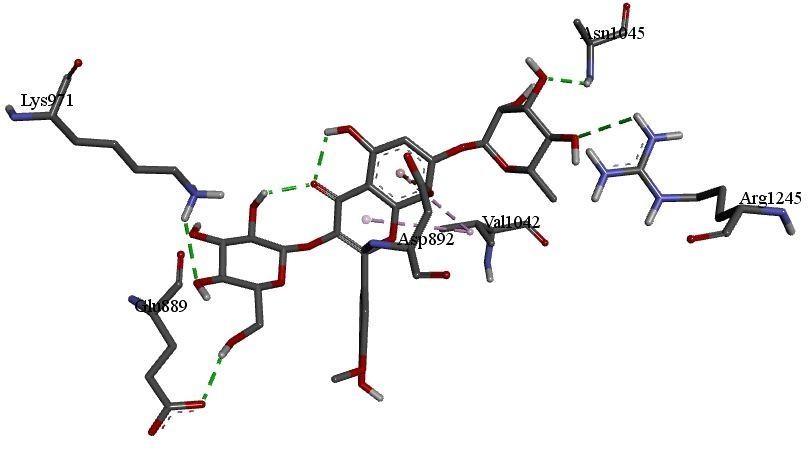


**Supplementary Table 3.** MMGB-SA binding free energies values (kcal/mol) for CYC and RAS1 Hits

| **Protein-** | **1MMGBSA** | **2MMGBSA** | **3MMGBSA** | **4MMGBSA** | **5MMGBSA** |
| --- | --- | --- | --- | --- | --- |
| **Ligand complex** | **Δ Bind** | **ΔCoul** | **ΔHbond** | **ΔLipo** | **ΔvdW** |
| **CYC-Farnesol** | -66.004 | -0.4057 | -0.0627 | -43.680 | -42.043 |
| **CYC-IGR** | -53.805 | -19.485 | -3.770 | -32.031 | -44.945 |
| **CYC-KCG** | -54.159 | -30.229 | -2.390 | -27.012 | -50.652 |
| **RAS-Farnesol** | -49.478 | -6.597 | -0.231 | -32.502 | -24.524 |
| **RAS-IGR** | -87.072 | -27.780 | -1.680 | -37.809 | -62.108 |
| **RAS-KCG** | -127.125 | -51.887 | -2.460 | -47.087 | -59.750 |

1 Binding free energy ; ^2^ Coulombic energy ; ^3^ Hydrogen bond energy ; ^4^ Lipophilic energy ; ^5^

Van der Waal energy.

**Supplementary Table 4.** Physicochemical Properties of Screened Natural Compounds

| **Ligands** | **Molecular** | **H-bond** | **H-** | **Fractio** | **Rotatable** | **Log** | **TPSA** | **Lipinski’s** |
| --- | --- | --- | --- | --- | --- | --- | --- | --- |
|  | **weight (g/mol)** | **acceptors** | **bonds donors** | **n Csp3** | **bonds** | **P** | **(Å²)** | **rule** |
| **KCG** | 594.52 | 13 | 7 | 0.20 | 8 | 2.93 | 216.58 | No; 3 |
|  |  |  |  |  |  |  |  | violations |
| **IGR** | 624.54 | 16 | 9 | 0.46 | 7 | 3.34 | 258.43 | No; 3 |
|  |  |  |  |  |  |  |  | violations |
| **Quercetin-3-** | 434.35 | 11 | 7 | 0.25 | 3 | 1.57 | 190.28 | No; 2 |
| **O-** |  |  |  |  |  |  |  | violations |
| **arabinoside** |  |  |  |  |  |  |  |  |
| **N1,N5,N10-** | 640.77 | 7 | 5 | 0.27 | 21 | 3.87 | 156.43 | Yes; 1 |
| **Tris-trans-p-** |  |  |  |  |  |  |  | violation: |
| **coumaroylsp** |  |  |  |  |  |  |  |  |
| **ermine** |  |  |  |  |  |  |  |  |
| **Acacetin** | 284.26 | 5 | 2 | 0.06 | 2 | 2.56 | 79.90 | Yes ; 0 |
|  |  |  |  |  |  |  |  | violation |
| **Luteolin 7-** | 534.42 | 14 | 7 | 0.29 | 8 | 1.58 | 233.65 | No; 3 |
| **O-(6 ''-** |  |  |  |  |  |  |  | violations |
| **malonylgluc** |  |  |  |  |  |  |  |  |
| **oside)** |  |  |  |  |  |  |  |  |
| **3,4’,5-** | 360.31 | 8 | 3 | 0.17 | 4 | 2.58 | 118.59 | Yes; 0 |
| **Trihydroxy-** |  |  |  |  |  |  |  | violation |
| **3’,6,7-** |  |  |  |  |  |  |  |  |
| **trimethoxyfl** |  |  |  |  |  |  |  |  |
| **avone** |  |  |  |  |  |  |  |  |
| **Genistein** | 270.24 | 5 | 3 | 0.00 | 1 | 1.91 | 90.90 | Yes ; 0 |
|  |  |  |  |  |  |  |  | violation |
| **Pinocembrin** | 256.25 | 4 | 2 | 0.13 | 1 | 2.11 | 66.76 | Yes ; 0 |
|  |  |  |  |  |  |  |  | violation |
| **Ferulic acid** | 194.18 | 4 | 2 | 0.10 | 3 | 1.62 | 66.76 | Yes ; 0 |
|  |  |  |  |  |  |  |  | violation |
